# HECTD3 inhibits NLRP3 inflammasome assembly and activation by blocking NLRP3-NEK7 interaction

**DOI:** 10.1038/s41419-024-06473-4

**Published:** 2024-01-24

**Authors:** Zhuo Cheng, Maobo Huang, Wei Li, Lei Hou, Li Jin, Qijin Fan, Linqiang Zhang, Chengbin Li, Li Zeng, Chuanyu Yang, Bin Liang, Fubing Li, Ceshi Chen

**Affiliations:** 1 Kunming Institute of Zoology, Chinese Academy of Sciences, Kunming, 650201 China; 2 Kunming College of Life Sciences, University of Chinese Academy Sciences, Kunming, 650204 China; 3 The First People’s Hospital of Kunming City & Calmette Affiliated Hospital of Kunming Medical University, Kunming, 650032 Yunnan China; 4 Department of Breast Disease, Henan Breast Cancer Center, Affiliated Cancer Hospital of Zhengzhou University & Henan Cancer Hospital, Zhengzhou, 450008 China; 5 The First Affiliated Hospital, Kunming Medical University, Kunming, 650032 China; 6 College of Life Sciences, Yunnan University, Kunming, 650500 China; 7 Academy of Biomedical Engineering, Kunming Medical University, Kunming, 650500 China; 8 The Third Affiliated Hospital, Kunming Medical University, Kunming, 650118 China

**Keywords:** Cell death and immune response, Immunological disorders

## Abstract

The NLRP3 inflammasome plays an important role in protecting the host from infection and aseptic inflammation, and its regulatory mechanism is not completely understood. Dysregulation of NLRP3 can cause diverse inflammatory diseases. HECTD3 is a E3 ubiquitin ligase of the HECT family that has been reported to participate in autoimmune and infectious diseases. However, the relationship between HECTD3 and the NLRP3 inflammasome has not been well studied. Herein, we show that HECTD3 blocks the interaction between NEK7 and NLRP3 to inhibit NLRP3 inflammasome assembly and activation. In BMDMs, *Hectd3* deficiency promotes the assembly and activation of NLRP3 inflammasome and the secretion of IL-1β, while the overexpression of HECTD3 inhibits these processes. Unexpectedly, HECTD3 functions in an E3 activity independent manner. Mechanically, the DOC domain of HECTD3 interacts with NACHT/LRR domain of NLRP3, which blocks NLRP3-NEK7 interaction and NLRP3 oligomerization. Furthermore, HECTD3 inhibits monosodium urate crystals (MSU)-induced gouty arthritis, a NLRP3-related disease. Thus, we reveal a novel regulatory mechanism of NLRP3 by HECTD3 and suggest HECTD3 could be a potential therapeutic target for NLRP3-dependent pathologies.

## Introduction

NOD-like receptor (NLR) family pyrin domain-containing 3 (NLRP3) is an important intracellular sensor that recognizes a broad range of PAMPs and DAMPs [1, 2]. NLRP3 protein is composed of three domains: a carboxy-terminal leucine-rich repeat (LRR) domain that can autoinhibit signal recognition capacities; a central nucleotide-binding domain (NACHT) that has ATPase activity and mediates self-oligomerization; and an amino-terminal pyrin domain (PYD) that can recruit apoptosis-associated speck-like protein containing a CARD (ASC) [3].

In the inactivate stage, LRR domain binds with NACHT domain and forms a self-inhibition protein conformation [4]. Upon activation, LRR domain of NLRP3 binds with NEK7 (Never in mitosis A-related kinase 7), and release NACHT domain [4]. Then, homotypic NACHT domain mediates the interaction of multiple NLRP3 proteins and triggers NLRP3 oligomerization. Oligomerized NLRP3 recruits ASC through PYD–PYD domain interactions and induces the oligomerization of ASC as well as the formation of ASC specks. Then, the oligomerized ASC recruits pro-caspase-1 through CARD–CARD domain interactions and forms the NEK7-NLRP3–ASC–pro-caspase-1 protein complex, which is named the NLRP3 inflammasome [5, 6].

The activation of NLRP3 inflammasome is triggered by stimuli from PAMPs or DAMPs and relies on two signals. First, TLR ligands (signal 1/priming signal) induce NLRP3 expression through the NF-κB pathway, as the protein level of NLRP3 is relatively low in resting macrophages [7]. Then, specific NLRP3 activators (signal 2/activating signal), such as extracellular adenosine triphosphate (ATP) [8], MSU crystals [9], amyloid-β [10], nigericin [8], and alum crystals [11] can trigger the assembly of the NLRP3 inflammasome. The assembled NLRP3 inflammasome can induce the self-cleavage and activation of pro-caspase-1, which results in the cleavage and maturation of pro-interleukin-1β (pro-IL-1β) and pro-interleukin-18 (pro-IL-18) [12]. In addition, activated caspase-1 also cleaves gasdermin D (GSDMD), which induces pyroptosis and mediates the release of cellular contents, including mature IL-1β, IL-18 and LDH [13].

Activation of the NLRP3 inflammasome is crucial for host defense against pathogen invasion and maintaining homeostasis [2]. However, excessive activation of the NLRP3 inflammasome also contributes to the progression of various inflammatory diseases, such as cryopyrin-associated periodic syndrome [14], arthritis [15], atherosclerosis [16], type 2 diabetes [17] and Alzheimer’s disease [18]. Therefore, activation of the NLRP3 inflammasome must be strictly regulated, and the mechanism of its activation also needs to be further investigated. Thus, regulation of NLRP3 expression, assembly, and activation offers an opportunity to alter the inflammatory potential of immune cells.

HECTD3 is a E3 ubiquitin ligase of the HECT family that plays critical roles in several inflammatory disease, including inflammation-related tumor metastasis [19], bacterial infection [20], RNA virus infection [21] and experimental autoimmune encephalomyelitis [22] etc., but its contribution to NLRP3 inflammasome remains unknown. Here, we reveal a critical role of HECTD3 in restraining NLRP3 inflammasome activation. As NLRP3 is ubiquitinated under resting conditions, and ubiquitination is important for NLRP3 inflammasome assembly and activation [23], We first hypothesized that HECTD3 may regulate NLRP3 inflammasome assembly and activation through regulating NLRP3 ubiquitination. However, HECTD3 negatively regulated the NLRP3 inflammasome assembly and activation in an E3 activity independent manner. Mechanistically, we found HECTD3 could interact with NLRP3, which can block the interactions between NLRP3 and NEK7. The destruction of their interactions could inhibit NLRP3 oligomerization and inflammasome assembly. Thus, HECTD3 inhibits the assembly and activation of the NLRP3 inflammasome by blocking NLRP3-NEK7 interaction and HECTD3 may be a promising target for treating NLRP3 inflammasome-related inflammatory diseases.

## Results

### *Hectd3* deficiency promotes the activation of NLRP3 inflammasome

**Fig. 1:**
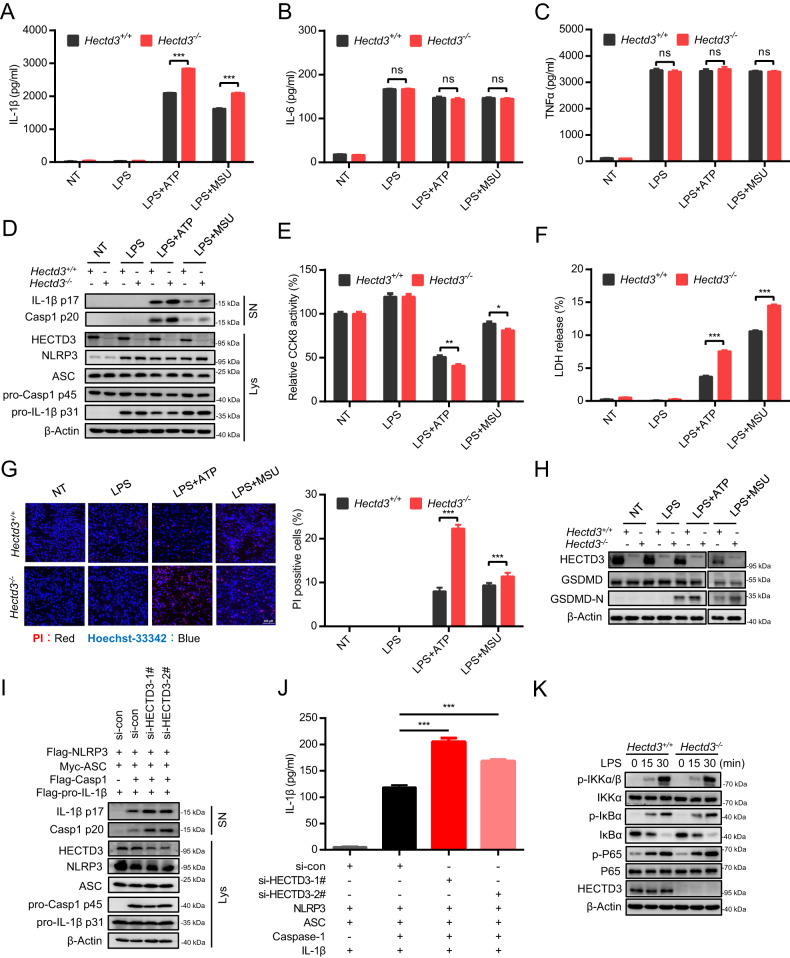
*Hectd3* deficiency promotes the activation of NLRP3 inflammasome. **A**–**H** BMDMs isolated from *Hectd3*^*+/+*^ and *Hectd3*^−/−^ mice were primed with LPS and stimulated with different secondary signals, including ATP and MSU. Supernatant IL-1β, IL-6 and TNF-α were analyzed using ELISA, *n* = 3 per group (**A**–**C**). Supernatants (SN) and cell extracts lysate (Lys) were analyzed using immunoblot assays (**D**). Cell death were detected by CCK8 asssay, *n* = 4 per group (**E**). Supernatants were collected to measure cell death by LDH release assay, *n* = 3 per group (**F**). PI staining was detected by fluorescence and the quantification of PI-positive cells is shown, *n* = 3 per group (**G**). The cleavage of GSDMD were detected using immunoblot assays (**H**); **I**, **J**. HEK293T cells were transfected with Flag-NLRP3, Myc-ASC, Flag-pro-caspase-1, Flag-pro-IL-1β plasmids as well as si-RNA of *HECTD3* and stimulated with 2.5 mM ATP. Supernatants (SN) and cell extracts lysate (Lys) were analyzed using immunoblot assays (**I**); Supernatant IL-1β were analyzed using ELISA, *n* = 3 per group (**J**); **K** BMDMs isolated from *Hectd3*^*+/+*^ and *Hectd3*^−/−^ mice were treated with 200 ng/mL LPS for 15 or 30 min, cell extracts were analyzed with immunoblot assays. Data are the mean ± SEM, ns (non-significant), *P* > 0.05; *P < 0.05; **P < 0.01; ***P < 0.001, two-tailed unpaired Student’s *t* test was used.

To determine whether HECTD3 affects NLRP3 inflammasome activation, we treated LPS-primed bone marrow-derived macrophages (BMDMs) isolated from WT (*Hectd3*^*+/+*^) and *Hectd3* KO (*Hectd3*^−/−^) mice with ATP and monosodium urate crystals (MSU), two different NLRP3 agonists. ELISA assays showed that *Hectd3* deficiency significantly promoted IL-1β secretion in both ATP or MSU stimulated BMDM cells, whereas no effect on the TNF-α or IL-6 secretion was observed (Fig. 1A–C). Immunoblot assay showed that *Hectd3* deficiency promoted the cleavage of pro-Caspase-1 and pro-IL-1β (Fig. 1D). In addition, *Hectd3* deficiency promotes NLRP3-mediated pyroptosis, as decreased CCK8 activity, increased LDH release rates and increased PI-positive cells were detected in *Hectd3*^−/−^ BMDMs under LPS plus ATP or LPS plus MSU treatment (Fig. 1E–G). *Hectd3* deficiency promoted the cleavage of pro-GSDMD as well (Fig. 1H). Furthermore, we reconstituted the NLRP3 inflammasome in HEK293T cells and observed that the *HECTD3* knockdown enhanced Caspase-1 cleavage and IL-1β secretion as well (Fig. 1I, J).

In our previous study, we reported that HECTD3 could promote the activation of NF-κB in HUVEC through promoting the ubiquitination of IKKα [19]. However, the secretions of IL-6 and TNF-α and the protein levels of NLRP3 and pro-IL-1β were not affected by HECTD3 under different stimulation conditions (Fig. 1B, C, D), indicating that HECTD3 does not affect the NF-κB pathway and the priming stage of the NLRP3 inflammasome under these conditions in BMDMs. To further confirm these results, we examined the activation of NF-κB under LPS treatment and found that *Hectd3* deficiency in macrophages did not mediate the phosphorylation of IKKα, IκBα and p65 under LPS treatment (Fig. 1K). Thus, we conclude that *Hectd3* deficiency promotes the activation but not the priming of NLRP3 inflammasome.

### HECTD3 inhibits the activation of NLRP3 inflammasome in an E3 activity independent manner

**Fig. 2:**
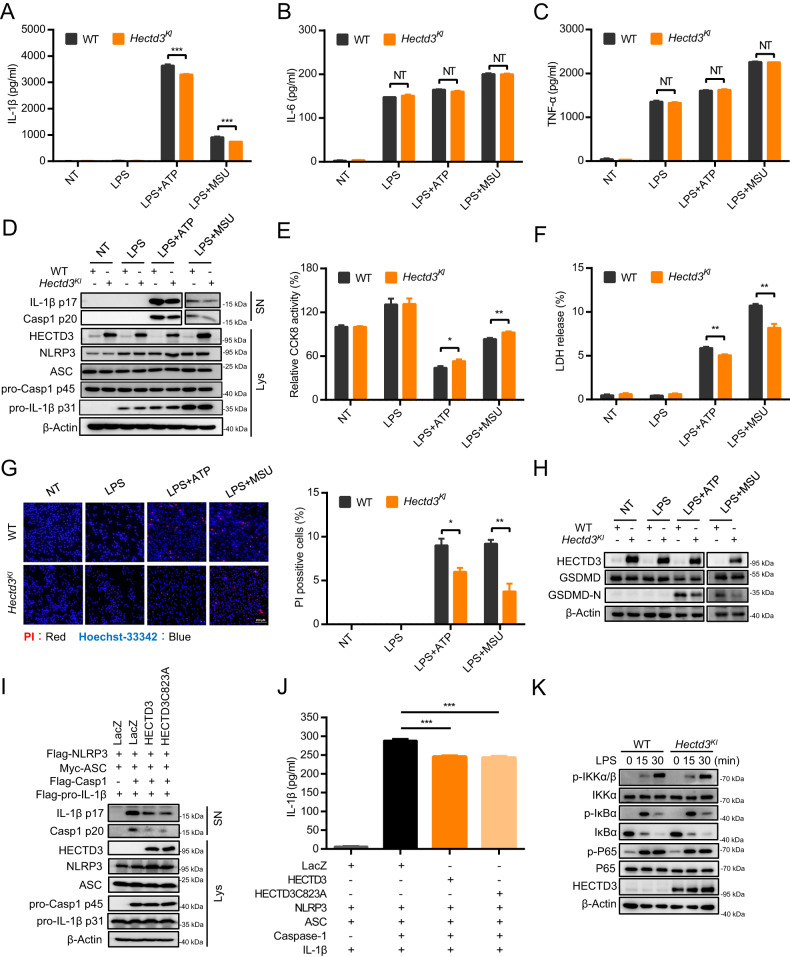
HECTD3 inhibits the activation of NLRP3 inflammasome in an E3 activity independent manner. **A**–**H** BMDMs isolated from WT and *Hectd3*^*KI*^ mice were primed with LPS and stimulated with different secondary signals, including ATP and MSU. Supernatant IL-1β, IL-6 and TNF-α were analyzed using ELISA, *n* = 3 per group (**A**–**C**). Supernatants (SN) and cell extracts lysate (Lys) were analyzed using immunoblot assays (**D**). Cell death were detected by CCK8 asssay, *n* = 4 per group (**E**). Supernatants were collected to measure cell death by LDH release assay, *n* = 3 per group (**F**). PI staining was detected by fluorescence and the quantification of PI-positive cells is shown, *n* = 3 per group (**G**). The cleavage of GSDMD were detected using immunoblot assays (**H**); **I**, **J** HEK293T cells were transfected with Flag-NLRP3, Myc-ASC, Flag-pro-caspase-1, Flag-pro-IL-1β plasmids as well as HECTD3 overexpression plasmid and stimulated with 2.5 mM ATP. Supernatants (SN) and cell extracts lysate (Lys) were analyzed using immunoblot assays (**I**); Supernatant IL-1β were analyzed using ELISA, *n* = 3 per group (**J**); **K** BMDMs isolated from WT and *Hectd3*^*KI*^ mice were treated with 200 ng/mL LPS for 15 or 30 min, cell extracts were analyzed with immunoblot assays. Data are the mean ± SEM, ns (non-significant), *P* > 0.05; *P < 0.05; **P < 0.01; ***P < 0.001, two-tailed unpaired Student’s *t* test was used.

To further address the role of HECTD3 in NLRP3 inflammasome activation, BMDMs isolated from WT and *Hectd3* transgenic (*Hectd3*^*KI*^) mice were treated with LPS plus ATP or MSU. As expected, ELISA assays showed that *Hectd3* overexpression significantly inhibited IL-1β secretion in both ATP or MSU stimulated BMDM cells, whereas no effects on the TNF-α or IL-6 secretion were observed (Fig. 2A–C). Moreover, higher cell viability, lower LDH release rates and fewer PI-positive cells were detected in *Hectd3*^*KI*^ BMDMs under LPS plus ATP or MSU treatment (Fig. 2E, G). The cleavage of pro-GSDMD, pro-Caspase-1 as well as pro-IL-1β were inhibited by *Hectd3* overexpression in ATP or MSU stimulated BMDM cells (Fig. 2D, H).

Since ubiquitination modification is important for NLRP3 inflammasome activation [23], we wondered whether HECTD3 inhibits NLRP3 activation through its E3 activity and we overexpressed HECTD3 WT or HECTD3C823A (a catalytically inactive mutant) in NLRP3 inflammasome-reconstituted HEK293T cells. Unexpectedly, the inhibitory effects of HECTD3 on NLRP3 inflammasome activation did not depend on its E3 activity, as the overexpression of HECTD3C823A inhibited Caspase-1 cleavage and IL-1β secretion as well as HECTD3 WT (Fig. 2I, J).

As the secretions of IL-6 and TNF-α, the protein levels of NLRP3 and pro-IL-1β and the activation of NF-κB signal remained largely unaffected by HECTD3 overexpression (Fig. 2B–D, K). We considered that HECTD3 inhibits the activation but not the priming of NLRP3 inflammasome and HECTD3 inhibits NLRP3 inflammasome activation in an E3 activity independent manner.

### *Hectd3* deficiency promotes the assembly of the NLRP3 inflammasome through enhancing NLRP3-NEK7 interaction

**Fig. 3:**
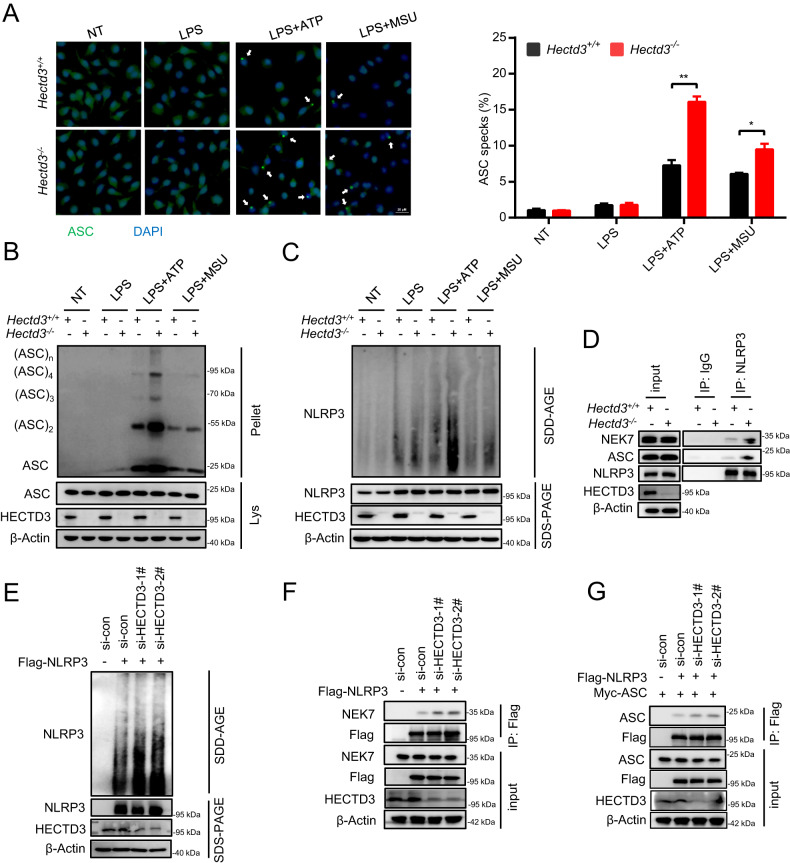
*Hectd3* deficiency promotes the assembly of the NLRP3 inflammasome through enhancing NLRP3-NEK7 interaction. **A**–**C**. BMDMs isolated from *Hectd3*^*+/+*^ and *Hectd3*^−/−^ mice were primed with LPS and stimulated with different secondary signals, including ATP and MSU. ASC speck formation was detected by immunofluorescence, and the quantification of ASC speck formation is shown, *n* = 3 per group (**A**); ASC oligomerization in cross-linked cytosolic pellets was detected by immunoblot assays (**B**); NLRP3 oligomerization in cytosolic pellets were analyzed with SDD–AGE assays. **D** BMDMs isolated from *Hectd3*^*+/+*^ and *Hectd3*^−/−^ mice were treated with LPS plus ATP, the interaction of NLRP3-NEK7 and NLRP3-ASC were detected by immunoprecipitation and immunoblot analysis. **E**, **F** HEK293T cells were transfected with Flag-tagged NLRP3 and *HECTD3* was knocked down using siRNA. The cells were treated with 2.5 mM ATP. NLRP3 oligomerization in cytosolic pellets were analyzed with SDD–AGE assays (**E**); The interactions of NLRP3-NEK7 were detected by immunoprecipitation and immunoblot analysis (**F**). **G** HEK293T cells were transfected with Flag-tagged NLRP3 and Myc-tagged ASC and *HECTD3* was knocked down using siRNA. The interaction of NLRP3-ASC was detected by immunoprecipitation and immunoblot assays (**G**). Data are the mean ± SEM, ns (non-significant), *P* > 0.05; *P < 0.05; **P < 0.01; ***P < 0.001, two-tailed unpaired Student’s *t* test was used.

Since HECTD3 inhibits the activation but not the priming of the NLRP3 inflammasome, we further hypothesized that HECTD3 inhibits the activation of the NLRP3 inflammasome by inhibiting its assembly. The assembly of the NLRP3 inflammasome could be reflected by ASC speck formation and ASC oligomerization. We found increased ASC specks formation and ASC oligomerization in *Hectd3*^−/−^ BMDMs under LPS plus ATP or MSU treatment (Fig. 3A, B).

NEK7-NLRP3 interaction, NLRP3 oligomerization and ASC recruitment to the NLRP3 oligomer are upstream events that precede ASC speck formation and ASC oligomerization. We wondered whether HECTD3 inhibits ASC speck formation and ASC oligomerization through mediating these steps. Thus, we first tried to define whether HECTD3 regulates NLRP3 oligomerization. Using a semidenaturing detergent agarose-gel electrophoresis (SDD–AGE) assay, we found that NLRP3 oligomerization was significantly enhanced in *Hectd3*^−/−^ BMDMs under NLRP3 inflammasome activation (Fig. 2C). In addition, NEK7-NLRP3 interaction and NLRP3–ASC interaction were markedly increased in *Hectd3*^−/−^ BMDMs under NLRP3 inflammasome activation (Fig. 2D). To further confirm these results, we transfected Flag-NLRP3 into HEK293T cells and interfered the expression of *HECTD3* with siRNA. The results showed that NLRP3 oligomerization were enhanced i*n HECTD3* knockdown HEK293T cells (Fig. 2E). Besides, the interaction between NEK7-NLRP3 and NLRP3-ASC were enhanced i*n HECTD3* knockdown HEK293T cells as well (Fig. 2F, G). Taken together, *Hectd3* deficiency promotes the assembly of the NLRP3 inflammasome through increasing the NLRP3-NEK7 interaction.

### HECTD3 inhibits the assembly of NLRP3 inflammasome through blocking NLRP3-NEK7 interaction in an E3 activity independent manner

**Fig. 4:**
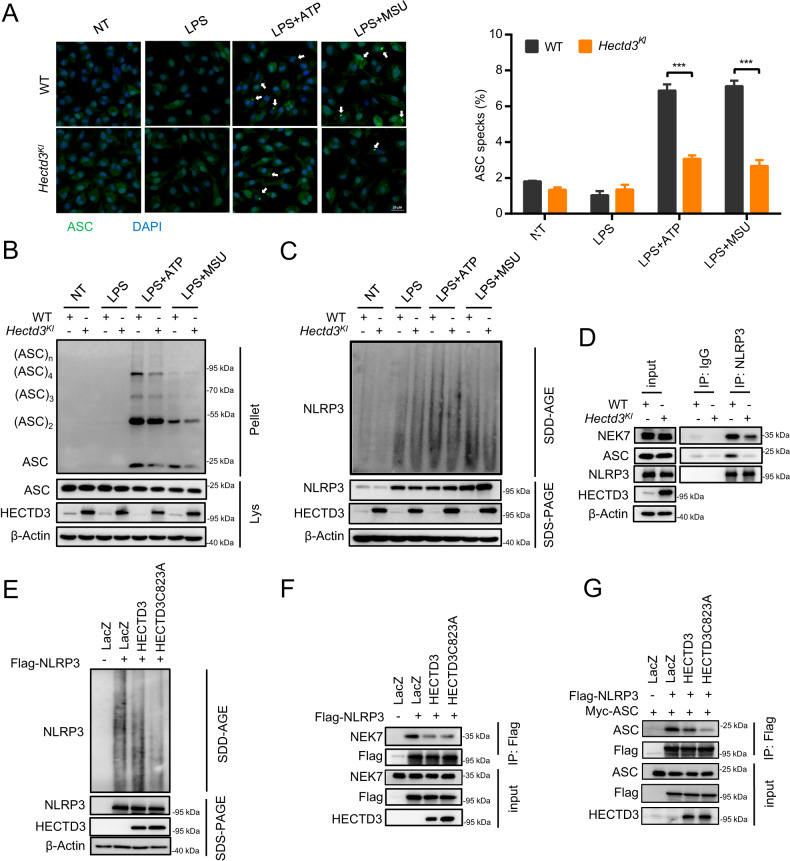
HECTD3 inhibits the assembly of NLRP3 inflammasome through blocking NLRP3-NEK7 interaction in an E3 activity independent manner. **A**–**C**. BMDMs isolated from WT and *Hectd3*^*KI*^ mice were primed with LPS and stimulated with different secondary signals, including ATP and MSU. ASC speck formation was detected by immunofluorescence, and the quantification of ASC speck formation is shown, *n* = 3 per group (**A**); ASC oligomerization in cross-linked cytosolic pellets was detected by immunoblot assays (**B**); NLRP3 oligomerization in cytosolic pellets were analyzed with SDD–AGE assays. **D** BMDMs isolated from WT and *Hectd3*^*KI*^ mice were treated with LPS plus ATP, the interaction of NLRP3-NEK7 and NLRP3-ASC were detected by immunoprecipitation and immunoblot assays. **E**, **F** HEK293T cells were transfected with Flag-tagged NLRP3 and HECTD3 WT or HECTD3C823A, and were treated with 2.5 mM ATP. NLRP3 oligomerization in cytosolic pellets were analyzed with SDD–AGE assays (**E**); The interactions of NLRP3-NEK7 were detected by immunoprecipitation and immunoblot assays (**F**). **G** HEK293T cells were transfected with Flag-tagged NLRP3, Myc-tagged ASC and HECTD3 WT or HECTD3C823A. The interaction of NLRP3-ASC was detected by immunoprecipitation and immunoblot assays (**G**). Data are the mean ± SEM, ns (non-significant), *P* > 0.05; *P < 0.05; **P < 0.01; ***P < 0.001, two-tailed unpaired Student’s *t* test was used.

As expected, ASC speck formation and ASC oligomerization were dramatically impaired in *Hectd3*^*KI*^ BMDMs under NLRP3 inflammasome activation (Fig. 4A, B). Besides, overexpression of HECTD3 inhibited NLRP3 oligomerization, NEK7-NLRP3 interaction and NLRP3–ASC interaction as well (Fig. 4C, D). These results further support that HECTD3 plays a negative regulatory role in the assembly of NLRP3 inflammasome. Using HEK293T transfected with indicate plasmids, we found the inhibitory effect of HECTD3 on NLRP3 inflammasome assembly did not dependent on its E3 activity, as expected (Fig. 4E–G).

Collectively, these results suggest that HECTD3 inhibits NEK7-NLRP3 interaction and NLRP3 oligomerization to disturb subsequent NLRP3-dependent ASC oligomerization and NLRP3 inflammasome assembly in an E3 ligase activity independent manner.

### The DOC domain of HECTD3 interacts with NACHT/LRR domain of NLRP3 and blocks NLRP3-NEK7 interaction

**Fig. 5:**
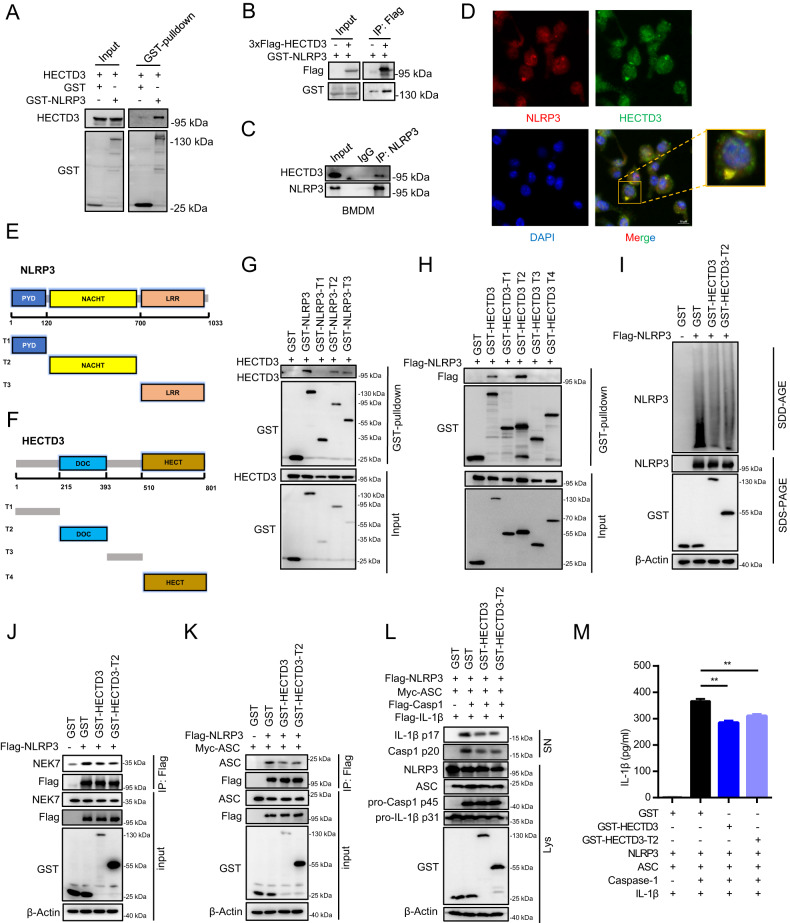
The DOC domain of HECTD3 interacts with NACHT/LRR domain of NLRP3 and blocks NLRP3-NEK7 interaction. **A** HECTD3 and GST-tagged NLRP3 were transfected into HEK293T cells. GST-tagged NLRP3 was pulled down and detected with the indicated antibodies; **B** GST-tagged NLRP3 and 3×Flag-tagged HECTD3 were transfected into HEK293T cells. Flag-tagged HECTD3 was immunoprecipitated and detected with the indicated antibodies; **C** BMDMs isolated from WT mice were treated with LPS plus ATP. Cell lysates were immunoprecipitated by anti-NLRP3 antibodies and detected with the indicated antibodies; **D** BMDMs isolated from WT mice were treated with LPS plus ATP. The colocalization of NLRP3 and HECTD3 was detected by immunofluorescence; **E** Schematic representation of NLRP3; **F** Schematic representation of HECTD3; **G** HECTD3 and GST-tagged NLRP3 or GST-tagged NLRP3 truncated mutants were transfected into HEK293T cells. GST-tagged NLRP3 or GST-tagged NLRP3 truncated mutants were pulled down and detected with the indicated antibodies; **H** 3×Flag-tagged NLRP3 and GST-tagged HECTD3 truncated mutants were transfected into HEK293T cells. GST-tagged truncated HECTD3 mutants were pulled down and immunoblotted with the indicated antibodies. **I**, **J** HEK293T cells were transfected with Flag-tagged NLRP3 and GST-HECTD3 or GST-HECTD3-DOC, and were treated with 2.5 mM ATP. NLRP3 oligomerization in cytosolic pellets were analyzed with SDD–AGE assays (**I**); The interactions of NLRP3-NEK7 were detected by immunoprecipitation and immunoblot assays (**J**). **K** HEK293T cells were transfected with Flag-tagged NLRP3, Myc-tagged ASC and GST-HECTD3 or GST-HECTD3-DOC. The interaction of NLRP3-ASC was detected by immunoprecipitation and immunoblot analysis (**K**). **L**, **M** HEK293T cells were transfected with Flag-NLRP3, Myc-ASC, Flag-pro-caspase-1, Flag-pro-IL-1β plasmids as well as GST-HECTD3 or GST-HECTD3-DOC, and were stimulated with 2.5 mM ATP. Supernatants (SN) and cell extracts lysate (Lys) were analyzed using immunoblot assay (**L**); Supernatant IL-1β were analyzed using ELISA, *n* = 3 per group (**M**). Data are the mean ± SEM, ns (non-significant), *P* > 0.05; *P < 0.05; **P < 0.01; ***P < 0.001, two-tailed unpaired Student’s *t* test was used.

HECTD3 is a member of the HECT family E3 ubiquitin ligases, which exhibit multiple functions by mediating the ubiquitination of target proteins [19, 20, 22]. However, the inhibitory effects of HECTD3 on NLRP3 inflammasome assembly and activation do not depend on its E3 ligase activity. We hypothesized that HECTD3 may interact with NLRP3 and block NLRP3- NEK7 interactions. We first detected the interactions between HECTD3 and NLRP3. GST-tagged NLRP3 and Flag-tagged HECTD3 were transfected into HEK293T cells. GST pull-down assays and IP assays showed that HECTD3 could interact with NLRP3 (Fig. 5A, B). Furthermore, we found that endogenous HECTD3 could interact with endogenous NLRP3 in BMDM as well (Fig. 5C). We also found colocalization of HECTD3 and NLRP3 in BMDM by confocal microscopy (Fig. 5D).

To further characterize the interaction of HECTD3 with NLRP3, we constructed a series of truncated mutants of HECTD3 and NLRP3 according to their domain structures (Fig. 5E, F). We found that both the NACHT and LRR domains of NLRP3 were important for its interaction with HECTD3 (Fig. 5G) and the DOC domain of HECTD3 was involved in its interaction with NLRP3 (Fig. 5H). Taken together, these results suggest that the DOC domain of HECTD3 and the NACHT/LRR domain of NLRP3 mediate the HECTD3-NLRP3 interactions.

As the LRR domain of NLRP3 mediates the interaction of NLRP3-NEK7, we hypothesized that HECTD3 may occupy the interaction area of NLRP3 and NEK7 through its DOC domain. To confirm this hypothesis, we overexpressed the DOC domain in HEK293T cells transfected with Flag-NLRP3, and we found the DOC domain itself could inhibit NLRP3 oligomerization and NEK7-NLRP3 interaction sufficiently (Fig. 4I, J). Moreover, NLRP3–ASC interaction and NLRP3 inflammasome activation were blocked by the DOC domain as well (Fig. 4K–M).

Collectively, these results suggest that the DOC domain of HECTD3 interacts with the NACHT/LRR domain of NLRP3 and the DOC domain can block NLRP3-NEK7 interaction, NLRP3 oligomerization, NLRP3-ASC interaction and NLRP3 inflammasome activation sequentially.

### HECTD3 inhibits NLRP3 inflammasome activation in *vivo*

**Fig. 6:**
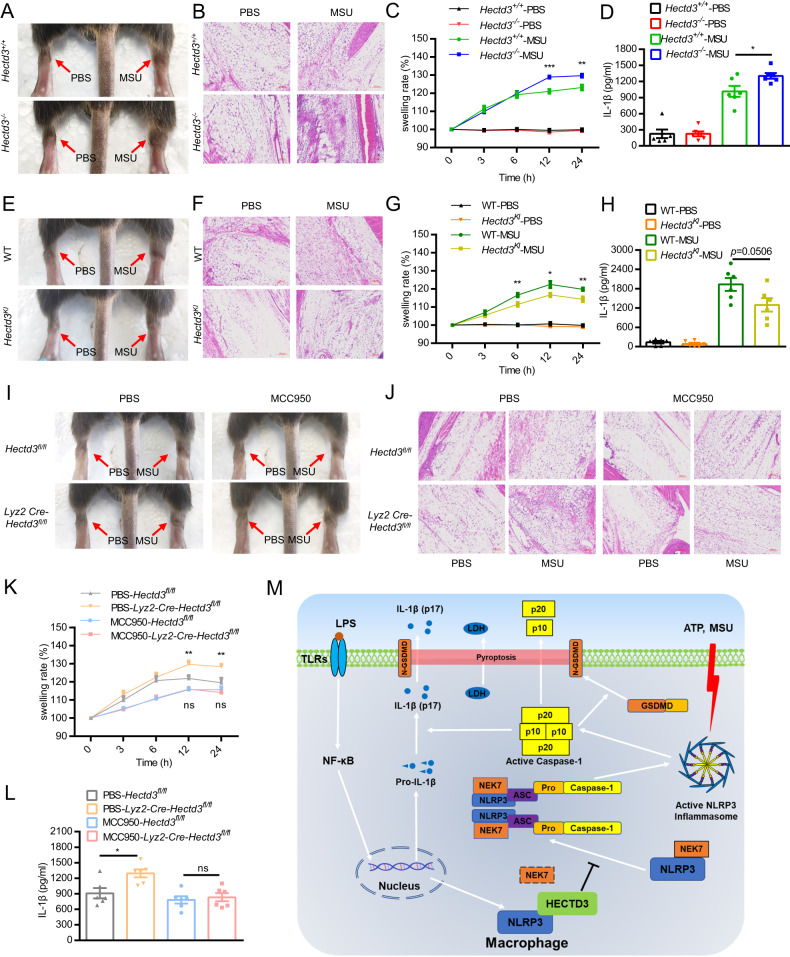
HECTD3 inhibits NLRP3 inflammasome activation in *vivo*. **A**–**D**. 1 mg of MSU crystals was injected into the ankle joint of *Hectd3*^*+/+*^ and *Hectd3*^−/−^ mice. After 24 h, the mice were sacrificed and the joints were pictured (**A**); Ankle joints from two mice were cut off and fixed for HE staining and imaging (**B**); The width of the ankle joint was measured by a Vernier caliper at each time. The joint swelling rate was expressed as a percentage of the primary width of the ankle joint, *n* = 8 mice per group (**C**); Ankle joints from six mice were cut off and tissue fragmentation were completely mixed with PBS, supernatant were collected and IL-1β levels were detected by ELISA, *n* = 6 mice per group. **E**–**H** 1 mg of MSU crystals was injected into the ankle joint of WT and *Hectd3*^*KI*^ mice. After 24 h, the mice were sacrificed and the joints were pictured (**E**); Ankle joints from two mice were cut off and fixed for HE staining and imaging (**F**); The width of the ankle joint was measured by a Vernier caliper at each time. The joint swelling rate was expressed as a percentage of the primary width of the ankle joint, *n* = 8 mice per group (**G**); Ankle joints from six mice were cut off and tissue fragmentation were completely mixed with PBS, supernatant were collected and IL-1β levels were detected by ELISA, *n* = 6 mice per group (**H**). **I**–**L**. 1 mg of MSU crystals was injected into the ankle joint of *Hectd3*^*fl/fl*^ and *Lyz2-Cre-Hectd3*^*fl/fl*^ mice, while PBS or MCC950 (10 mg/kg) were i.p. injected. After 24 h, the mice were sacrificed and the joints were pictured (**I**); Ankle joints from two mice were dissected and fixed for HE staining and imaging (**J**); The width of the ankle joint was measured by a Vernier caliper at each time. The joint swelling rate was expressed as a percentage of the primary width of the ankle joint, *n* = 8 mice per group (**K**); Ankle joints from six mice were cut off and tissue fragmentation were completely mixed with PBS, supernatant were collected and IL-1β levels were detected by ELISA, *n* = 6 mice per group (**L**). **M** Working model for this work. Data are the mean ± SEM, ns (non-significant), *P* > 0.05; *P < 0.05; **P < 0.01; ***P < 0.001, two-tailed unpaired Student’s *t* test was used.

Since HECTD3 inhibited NLRP3 inflammasome activation in *vitro*, we wondered whether HECTD3 would also suppress NLRP3-related inflammatory responses in *vivo*. Excessive NLRP3 inflammasome activation has been found to be closely related to a variety of human inflammatory diseases. Herein, MSU-induced gouty arthritis, an acute NLRP3-related inflammatory model was used to clarify the function of HECTD3 in *vivo*. In the MSU-induced gout model, mice were intra-articular injected with 1 mg of MSU to activate NLRP3 inflammasome-dependent arthritis. Consistent with previous studies, intra-articular injection of MSU caused acute joint swelling, immune cells infiltration, and IL-1β secretion in joint tissue. These effects were enhanced by HECTD3 deficiency and inhibited by *Hectd3* overexpression (Fig. 6A–H). We therefore confirmed that HECTD3 could inhibit NLRP3 inflammasome activation in *vivo*.

In order to demonstrate that HECTD3 inhibits NLRP3 activation through inhibiting NLRP3 inflammasome activation in myeloid cells, we generated myeloid-specific *Hectd3* knockout mice (*Lyz2-Cre-Hectd3*^*fl/fl*^) by crossing *Lyz2-Cre* mice with *Hectd3*^*fl/fl*^ mice. MSU-induced gouty arthritis was generated in conditional knockout mice (Lyz2-Cre-*Hectd3*^*fl/fl*^) and their WT littermates (*Hectd3*^*fl/fl*^), while MCC950, a NLRP3 inhibitor [24] was i.p. injected into these mice. Subsequently, we found that *Hectd3* deficiency in myeloid cells could promote MSU-induced gouty arthritis (Fig. 6I–L). Besides, inhibition of NLRP3 could block the extra inflammatory responses in conditional knockout mice (Fig. 6I–L). Collectively, these findings showed that HECTD3 in myeloid cells reduced MSU-induced gouty arthritis, a NLRP3-dependent disease via suppressing the activation of the NLRP3 inflammasome.

Altogether, these data demonstrate that HECTD3 directly interacts with NLRP3 via its DOC domain and this interaction blocks NLRP3-NEK7 interaction and inhibits subsequently NLRP3 inflammasome assembly and activation (Fig. 6H). This study demonstrates that HECTD3 plays a critical role in MSU-induced gouty arthritis, a NLRP3-related diseases and suggests potential therapeutic strategies for NLRP3-related inflammatory diseases by targeting the HECTD3-NLRP3 interaction.

## Discussion

NLRP3 is an innate immune sensor that can trigger an inflammatory cascade in response to a variety of intracellular and extracellular stresses, making NLRP3 an important role in a wide range of physiological processes including pathogens and injury. However, excessive activation of the NLRP3 inflammasome has been implicated in a wide variety of diseases, such as cryopyrin-associated periodic syndrome [14], arthritis [15], atherosclerosis [15], type 2 diabetes [17], Alzheimer’s disease [18], and cancer [25]. Thus, fine-tuning NLRP3 inflammasome activity is essential for preventing NLRP3-related diseases and maintaining immune homeostasis. NLPR3 inflammasome activation is regulated by multiple mechanisms at different stages. In the resting stage, the expression of NLRP3 is very low, which makes NLRP3 inflammasome assembly hardly induced. In the LPS-primed stage, NLRP3 expression is upregulated, but the ubiquitination of NLRP3 could limit its assembly and activity [26]. These multiple regulatory mechanisms allow NLRP3 inflammasome activation to be limited to an appropriate intensity and time course to avoid harmful effects. However, when confronted with an emergency, the NLRP3 inflammasome needs to be assembled and activated immediately to respond to possible hazards. Thus, NLRP3 activity may need to be fine-tuned to quickly respond to potential threats as well. Of particular note is the mitotic serine/threonine kinase NEK7, which has recently been identified as an important requirement for activation at NLRP3 through a direct interaction with NLRP3 [5, 6].

HECTD3 plays important roles in experimental autoimmune encephalomyelitis [22], bacterial infection [20] and inflammation-related tumor metastasis [19]. We demonstrate that HECTD3 inhibits gouty arthritis, a NLRP3-related inflammatory disease by inhibiting assembly and activation of the NLRP3 inflammasome. Noteworthily, previous studies had proposed a role for HECTD3 in LPS-induced NF-κB activation to promote adhesion molecule and inflammatory factor gene expression in vascular endothelial cells. However, HECTD3 is not involved in LPS-induced activation of NF-κB or induction of pro-IL-1β or NLRP3 in BMDM, but instead inhibits NLRP3 inflammasome assembly and activation. Notably, the inhibitory effects of HECTD3 on NLRP3 inflammasome assembly and activation do not depend on its E3 ligase activity. Mechanistic studies suggest that HECTD3 interact with NLRP3 directly, which blocks NLRP3-NEK7 interactions, NLRP3 oligomerization, NLRP3-ASC interaction and NLRP3 inflammasome activation sequentially. Furthermore, we found the DOC domain, which mediates HECTD3-NLRP3 interactions could inhibit NLRP3-NEK7 interactions, NLRP3 oligomerization, NLRP3-ASC interaction and NLRP3 inflammasome activation sufficiently.

Over the past decade, great efforts have been paid to develop small-molecule inhibitors of the NLRP3 inflammasome, some of which have shown remarkable therapeutic potential [24, 27–29]. However, we are still far from successful clinical applications of these inhibitors. Therefore, the development of novel efficient inhibitors for the NLRP3 inflammasome is of great importance. Recently, hundreds of short epitopes have been developed as inhibitors of protein-protein interactions and display therapeutic promise against numerous diseases [30]. In this study, we suggested a therapeutic strategy by precisely targeting the HECTD3-NLRP3 interaction, which could be applied to the clinical treatment of NLRP3-dependent diseases. Through fine mapping of the interaction sequence between HECTD3 and NLRP3, we could mimic a homologous peptide from HECTD3 that participate in HECTD3-NLRP3 interactions, which could be developed as a promising inhibitor for NLRP3-dependent diseases.

In conclusion, we revealed a previously unknown role for the E3 ubiquitin ligase HECTD3 in macrophages as an important suppressor of NLRP3 inflammasome activation in an E3 activity independent manner. We demonstrate that HECTD3 directly interacts with NLRP3 via its DOC domain, and the interaction between HECTD3 and NLRP3 blocks NLRP3-NEK3 interactions, thereby inhibiting NLRP3 inflammasome assembly and activation. Thus, this work identifies a novel regulatory mechanism for NLRP3 inflammasome activation and proposes HECTD3 as a new therapeutic target for NLRP3 inflammasome-associated diseases.

## Methods

### Antibodies and reagents

Anti-HECTD3 (Homemade, 1:1000), anti-IL-1β (R&D Systems, 1:1000, AF-401-NA), anti-NLRP3 (Adipogen, 1:1000, AG-20B-0014), anti-ASC (CST, 1:1000, 67824), anti-caspase-1 (p20) (Adipogen, 1:1000, AG-20B-0042), anti-caspase-1 (p10) (Adipogen, 1:1000, AG-20B-0044), anti-HA (Rockland, 1:2000, 600-401-384), anti-GST (Sigma, 1:5000, G7781), anti-FLAG (Sigma, 1:5000, F3165), anti-Actin (Sigma, 1:5000, A5441), HRP-linked anti-rabbit IgG (Sigma, 1:5000, 12-348), HRP-linked anti-mouse IgG (Sigma, 1:5000, 12-349) and HRP-linked anti-goat IgG (ABclonal, 1:5000, AS029) were used for Western blotting.

Anti-NLRP3 (Adipogen, 1:200, AG-20B-0014) and Mouse mAb IgG1 Isotype Control (CST, 1:200, 5415) were used for immunoprecipitation.

Anti-NLRP3 (Adipogen, 1:200, AG-20B-0014), anti-HECTD3 (Proteintech, 1:200, 11487-1-AP), anti-ASC (CST, 1:200, 67824), ABflo® 488-conjugated donkey anti-rabbit IgG (H + L) (ABclonal, 1:200, AS053) and ABflo® 594-conjugated donkey anti-mouse IgG (H + L) (ABclonal, 1:200, AS054) were used for immunofluorescence.

LPS and ATP were purchased from Sigma-Aldrich; Nigericin were purchased from MCE; Lipofectamine 2000 Transfection Reagent was purchased from Invitrogen; propidium iodide and Hoechst-33342 were purchased from Beyotime; anti-FLAG® M2 Magnetic Beads (M8823) were purchased Sigma-Aldrich; GST beads (10268095) were purchased from GE HEATHYCARE; Protein A/G PLUS-Agarose (sc-2003) were purchased from Santa Cruz Biotechnology.

### Mice

C57BL/6 mice were purchased from Hunan SJA Laboratory Animal Co., Ltd. *Hectd3*^−/−^ mice were generated by Taconic Farms, Inc. (TF2706, TACONIC KNOCKOUT REPOSITORY) with a 129/SvEv and C57BL/6 Chimeric background, which was mentioned in our previous study [20]. Chimeric mice were backcrossed to the C57BL/6 genetic background for eight generations. C57BL/6 background heterozygous (*Hectd3*^+/−^) mice were hybridized to obtain WT (*Hectd3*^*+/+*^) mice and homozygous (*Hectd3*^−/−^) mice, which were used in this study. The loxP-Stop-loxP-*Hectd3* CDS-GFP C57BL/6 strain mice (project number: EGE-ZLY-004 KI) were generated by Beijing Biocytogen Co., Ltd, as described in our previous study [19]. The loxP-Stop-loxP-*Hectd3* CDS-GFP mice were crossed with CMV-Cre mice to generate whole-body knock-in *(Hectd3*^*KI*^*)* mice. These *Hectd3*^*KI*^ mice and WT littermates were used in this study. The loxP-*Hectd3*-loxP C57BL/6 strain mice (project number: EGE-SSH-021-B) were generated by Beijing Biocytogen Co., Ltd as described in our previous study [19]. The loxP-*Hectd3*-loxP C57BL/6 strain mice were crossed with Lyz2-Cre mice to generate myeloid-specific *Hectd3* knockout mice (*Lyz2-Cre-Hectd3*^*fl/fl*^) These conditional knockout mice (Lyz2-Cre-*Hectd3*^*fl/fl*^) and their WT littermates (*Hectd3*^*fl/fl*^) were treated with MCC950 (10 mg/kg) and used for *vivo* assay. All mice were maintained in specific pathogen-free conditions at 20–26°C and 40–70% humidity on an 8 pm/8 am nocturnal dark/light cycle at the Animal Resource Center of Kunming Institute of Zoology, Chinese Academy of Sciences. All animal experiments were conducted in accordance with the guidelines and were approved by the Kunming Institute of Zoology, Chinese Academy of Sciences Animal Care and Use Committee. Eight- to twelve-week-old male mice were used for harvesting BMDMs and in *vivo* experiments. Animal experiments were approved by Academy of Biomedical Engineering, Kunming Medical University (kmmu20231321).

### Cell culture and treatments

HEK293T cells were cultured in Dulbecco’s modified Eagle’s medium (DMEM) with 5% fetal bovine serum and 1% penicillin/streptomycin. L929 cells were cultured in RPMI-1640 medium with 10% fetal bovine serum and 1% penicillin/streptomycin. HEK293T and L929 cells were obtained from our own laboratory and were tested negative for mycoplasma, bacteria, yeast and fungi according to the manufacturer. Primary bone marrow-derived cells were harvested as previously described [20]. Briefly, bone marrow cells were flushed from the tibias and femurs of mice and cultured in DMEM supplemented with 10% fetal bovine serum, 1% penicillin/streptomycin, and 20% L929 supernatant for 5–6 days to generate BMDMs. To obtain L929 supernatant, 2.5 × 10^6^ L929 cells were seeded in 225 T culture flasks and cultured for 7 days. The supernatant was harvested and filtered through a 0.45 μM filter membrane. After filtration, the L929 supernatant was stored at −80 °C for later use. For NLRP3 inflammasome activation, 2 × 10^6^ BMDMs were plated in 6-well plates overnight. The following day, cells were primed with LPS (200 ng/mL) for 4 h. After LPS priming, the cells were stimulated with 2.5 mM ATP for 30 min or 200 μg/mL MSU for 4 h to activate the NLRP3 inflammasome. To stimulate the assembly and activation of NLRP3 inflammasome in HEK293T, cells were stimulated with 2.5 mM ATP for 3 h. All cells were cultured at 37 °C with 5% CO_2_.

### ELISA

Mouse IL-1β, IL-6 and TNF-α levels in cell culture supernatants were quantitated using the mouse IL-1β ELISA Kit (Beijing 4 A Biotech-Cat No. CME0015), mouse IL-6 ELISA kit (Beijing 4 A Biotech-Cat No. CME0006) and mouse TNF-α ELISA Kit (Beijing 4 A Biotech-Cat No. CME0004) according to the manufacturer’s protocol. The standard curve was generated using the kit’s standards.

### Propidium iodide (PI) staining assay

Cells were seeded in chamber slides overnight. The following day, the cells were treated with the indicated conditions. PI (1 μg/mL) and Hoechst-33342 (0.5 μg/mL) were added to the chamber slides and incubated for 30 min at 37 °C. After piece sealing, the cells were imaged.

### Lactate dehydrogenase (LDH) release assay

Pyroptosis could be quantitated by analyzing the activity of LDH released into cell culture supernatants after various treatments. LDH activity was detected by the LDH Cytotoxicity Assay Kit (Beyotime-Cat No. C0017) according to the manufacturer’s protocols. The LDH activity in the culture supernatant was expressed as a percentage of total LDH in the cell lysate.

### Protein sample preparation and Western blot analysis

Supernatant protein: Supernatant proteins were precipitated by the methanol/chloroform method. Briefly, culture media were centrifuged at 2000 × *g* for 10 min to pellet cells and cell debris. Then, 600 μL of supernatant was transferred to a fresh tube, and 600 μL of methanol and 150 μL of chloroform were added. Samples were mixed well and centrifuged at 12,000 × *g* for 10 min. The upper phase was removed, and 600 μL methanol was added to each sample. Samples were mixed well and centrifuged at 12,000 × *g* for 10 min again. Supernatants were removed, and the remaining protein pellets were dried at 55 °C and resuspended in 2×SDS loading buffer. Samples were boiled for 10 min at 98 °C until dissolved.

Cell lysates: After stimulation with ATP or MSU, BMDMs were washed with PBS twice and lysed in 500 μL Triton X-100 lysis buffer (50 mM Tris-HCl pH7.6, 0.5% Triton X-100) and protease inhibitor cocktail on ice for 30 min. The cell lysates were centrifuged at 500 × *g* at 4 °C for 10 min. Then, 100 μL supernatants of lysate were collected for Western blot analysis, and others were removed. Precipitated pellets were used to detect ASC oligomerization and NLRP3 oligomerization as described below.

ASC oligomerization assays [31]: After lysed with Triton X-100 buffer, precipitated pellets were washed twice with PBS, resuspended with PBS containing 2 mM disuccinimidyl suberate (DSS) and incubated at room temperature for 30 min to crosslink ASC. Thirty minutes later, 1 mL Triton X-100 buffer was added into the sample and incubated at room temperature for 15 min to quench the crosslinking reaction. Samples were then centrifuged at 12,000 × *g* for 15 min, and the cross-linked pellets were resuspended with 60 µl 2×SDS loading buffer and boiled for 10 min at 98 °C. ASC oligomerization was analyzed by Western blotting.

NLRP3 oligomerization assays [32]: NLRP3 oligomerization was detected with semidenaturing detergent agarose gel electrophoresis (SDD-AGE). After lysed with Triton X-100 buffer, precipitated pellets were washed twice with PBS and resuspended with 1× sample buffer (0.5× TBE, 10% glycerol, 2% SDS and 0.0025% bromophenol blue). Samples were loaded onto a vertical 1.5% agarose gel. After electrophoresis in TBE running buffer (1× TBE and 0.1% SDS) for 1 h at a constant voltage of 100 V on ice, the proteins were transferred to PVDF membranes for 1 h at a constant voltage of 100 V on ice. PVDF membranes were blocked with 5% nonfat milk, and blots were probed with anti-NLRP3 antibodies.

Western blotting analysis: Except for the NLRP3 oligomerization assay, protein samples were separated by 11% SDS‒PAGE for 1 h at a constant voltage of 100 V, followed by electrophoretic transfer onto PVDF membranes for 1 h at a constant voltage of 100 V on ice. After that, PVDF membranes were blocked with 5% nonfat milk, and blots were probed with appropriate antibodies at 4 °C overnight. The following day, PVDF membranes were washed with PBST for 10 min 3 times and incubated with the corresponding HRP-linked secondary antibody.

### Immunofluorescence

BMDMs were seeded on chamber slides overnight. The following day, cells were treated with the indicated conditions and fixed in 4% paraformaldehyde in PBS at room temperature for 30 min. Fixed cells were permeabilized with 0.5% Triton X-100 in PBS for 5 min, rinsed with PBS twice, blocked with 5% BSA in PBS for 30 min and incubated with primary antibodies overnight at 4 °C. The following day, the cells were washed three times with PBST (0.05% Tween-20 in PBS) and incubated with the indicated fluorescence-labeled secondary antibodies at room temperature for 1 h. After extensive washing with PBST, the cells were stained with DAPI for 15 min. After piece sealing, the cells were imaged.

### Transfection

The coding region of human HECTD3 was cloned into pLenti6-Vector or PCDH-3×Flag-Vector. The coding regions of mouse NLRP3 and Caspase-1 were cloned into PCDH-3×Flag-Vector. Plasmids encoding HA-ubiquitin or ubiquitin mutants have been described previously. Plasmids were transfected into HEK293T cells using polyethyleneimine (PEI) or Lipofectamine 2000 Transfection Reagent.

The small interfering RNA sequence was as follows: Si-*Hectd3-*1#: 5′- GCG GGA ACU AGG GUU GAA Utt -3′; Si-*Hectd3-*2#: 5′GGU AUU UCA CCU CUU AAG Att -3′. Scramble siRNA was used as a negative control in all RNA interference experiments. These siRNAs were obtained from Guangzhou RiboBio Co., LTD and transfected into cells with Lipofectamine 2000 according to the manufacturer’s instructions.

### Immunoprecipitation (IP) and GST-pulldown

For Flag-IP or GST-Pulldown, transfected HEK293T cells were lysed with IP lysis buffer (50 mM Tris-HCl, 5 mM EDTA, 150 mM NaCl, 0.5% (vol/vol) Nonidet-P40 and 10% (vol/vol) glycerol, pH 7.4) and protease inhibitor cocktail for 30 min on ice. Cell lysates were immunoprecipitated with Flag-M2 beads or GST beads with rotation at 4 °C overnight. The following day, the beads were washed 5 times with IP lysis buffer, resuspended with 2×SDS loading buffer and analyzed by Western blotting.

For the endogenous IP assays, the lysates of BMDMs were incubated with 2 μg/mL antibody recognizing a particular protein or isotype-matched IgG at 4 °C overnight. 20 μL protein A/G beads were incubated with the sample for 1 h at 4 °C the following day. After washing 5 times with IP lysis buffer, the samples were resuspended with 2 × SDS loading buffer and analyzed by Western blotting.

### Reconstitution of the NLRP3 inflammasome in HEK293T cells

NLRP3 inflammasome reconstructed HEK293T system was established as described [33]. HEK293T cells (5 × 10^5^) were seeded into six-well plates overnight. The following day, plasmids expressing Flag-pro-caspase-1 (120 ng), Myc-ASC (300 ng), and Flag-NLRP3 (400 ng) were transfected. Besides, cells were transfected with siRNA to knockdown HECTD3 or transfected with plasmids to overexpress HECTD3. The medium was replaced 8 h after transfection. The cells were treated with ATP (2.5 mM) for 1 h before sample collection, and the activation of the NLRP3 inflammasome was analyzed by LDH release assays and Western blot.

### MSU-induced gout model

MSU crystals were prepared as described [34]. Briefly, 1.68 g of uric acid was added into 0.01 M NaOH and heated to 70 °C. HCl/NaOH was added to adjust the pH to 7.1 between 7.2, and the solution was cooled to RT. As the temperature drops, the MSU crystals can precipitate gradually. Twenty-four hours later, MSU crystals were harvested, washed, dried and dispensed into individual tubes for later use.

For MSU-induced gout model, 1 mg MSU crystals resuspended in 25 μL PBS were injected into the ankle joint of male mice. The width of the ankle joint was measured by a Vernier caliper at each time. The joint swelling rate was expressed as a percentage of the primary width of the ankle joint. After 24 h, the mice were sacrificed. The ankle joint was dissected and fixed for HE staining and imaging.

### Quantification and statistical analysis

Data was presented as the mean ± SEM. Statistical analyses of two groups were performed with a two-tailed unpaired Student’s *t* test (GraphPad Prime 6 software). None of the samples were excluded. The researchers were not blinded during sample collection or data analysis. Differences were considered significant when *p ≤ 0.05, **p ≤ 0.01, ***p ≤ 0.001.

### Supplementary information

checklist

Original Data File

## Data Availability

The authors confirm that the data supporting the findings of this study are available within the article [and/or] its supplementary materials.
