# Supplementary material for: HECTD3 inhibits NLRP3 inflammasome assembly and activation by blocking NLRP3-NEK7 interaction
Source: Cell Death Dis. 2024 Jan 24;15(1):86. doi: 10.1038/s41419-024-06473-4 (PMC10808187; doi:10.1038/s41419-024-06473-4)
Supplement: Supplementary file 2 — Original Data File [file 41419_2024_6473_MOESM2_ESM.pdf]

Fig.1D

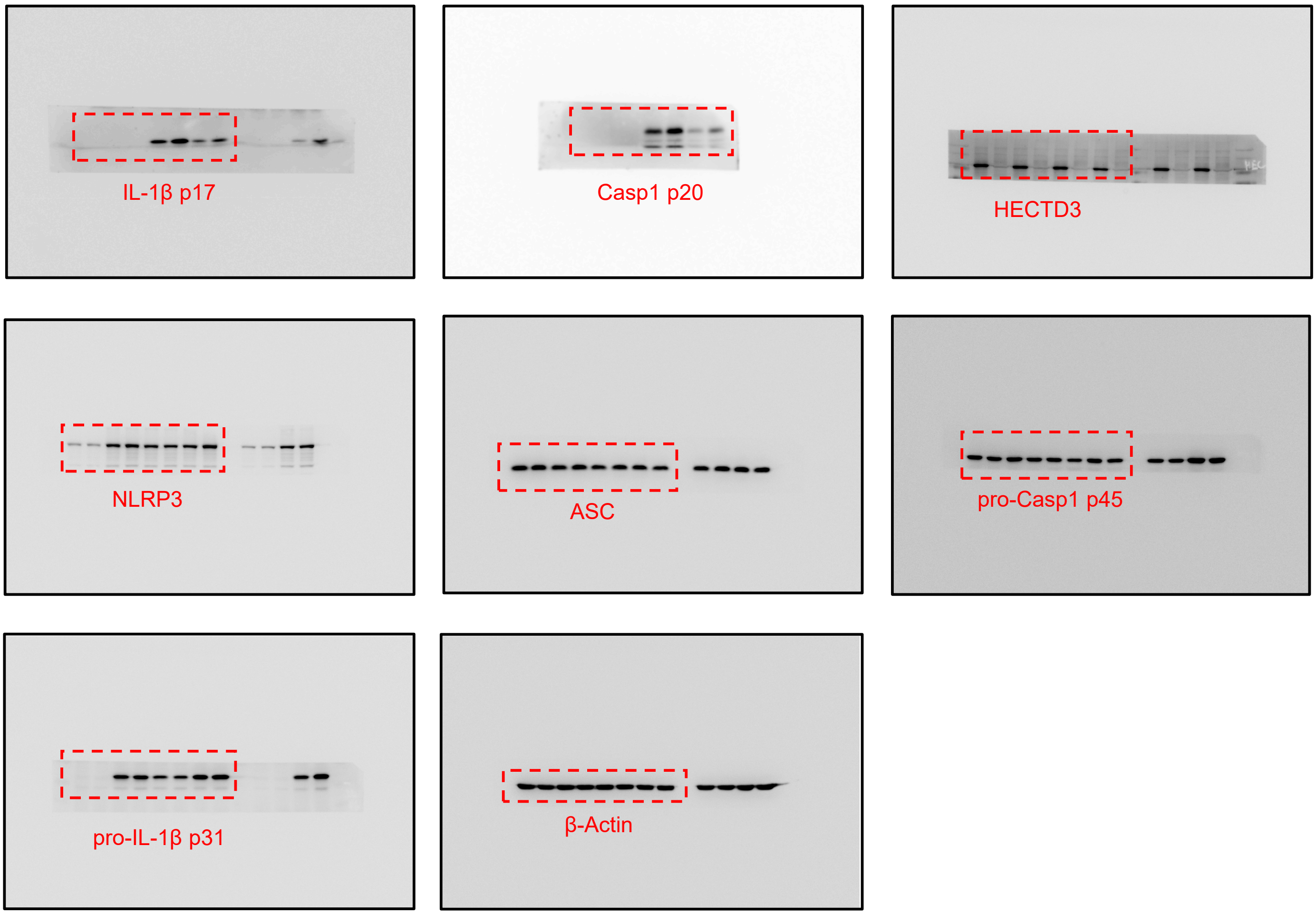
IL-1β p17
Casp1 p20
HECTD3
NLRP3
ASC
pro-Casp1 p45
pro-IL-1β p31
β-Actin

Fig.1H

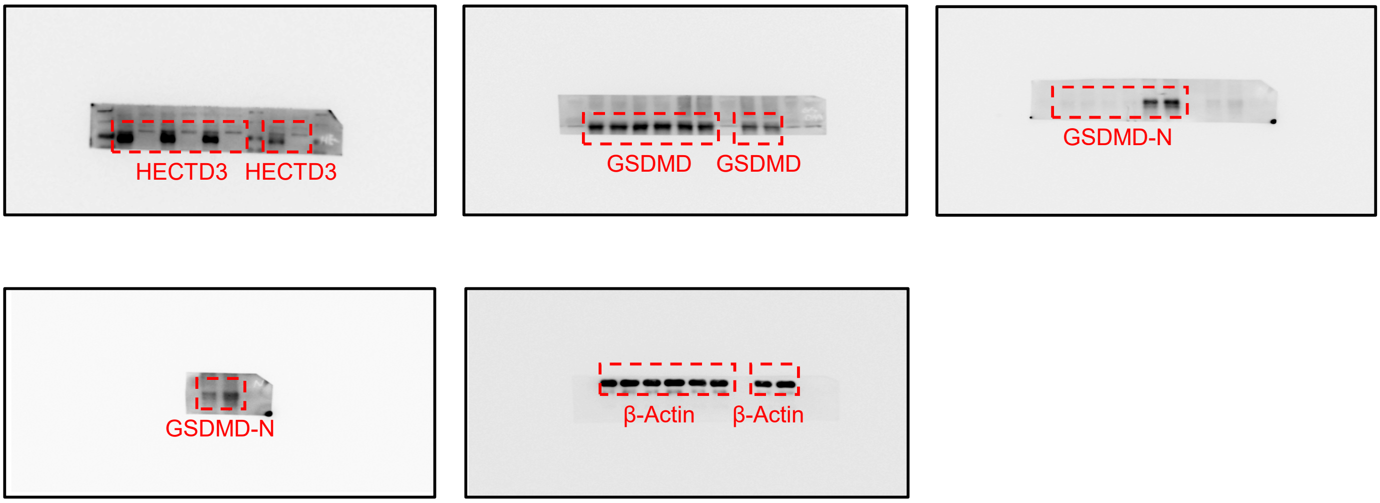
HECTD3
HECTD3
GSDMD
GSDMD
GSDMD-N
GSDMD-N
β-Actin
β-Actin

Fig.1G

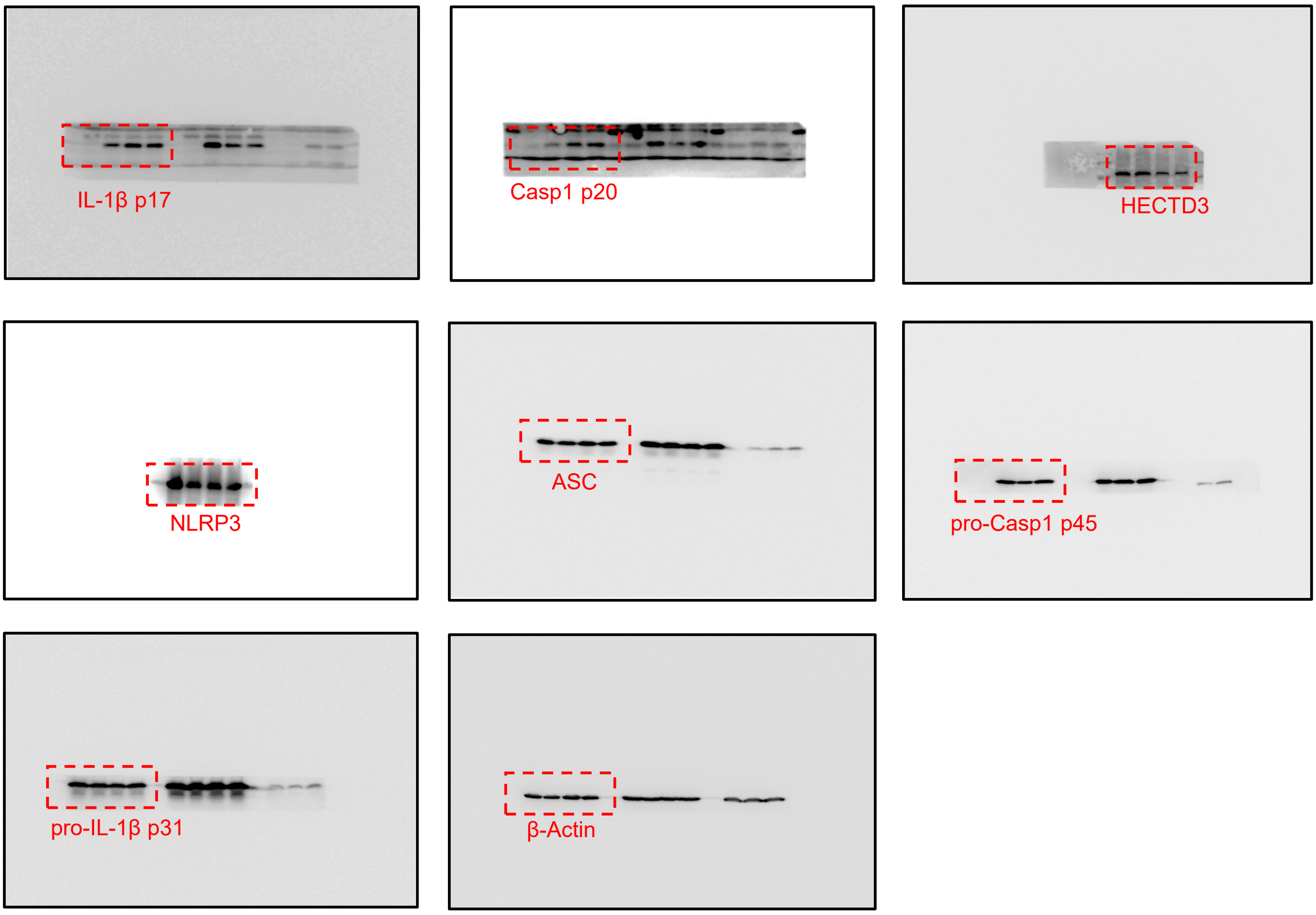
IL-1β p17
Casp1 p20
HECTD3
NLRP3
ASC
pro-Casp1 p45
pro-IL-1β p31
β-Actin

Fig.1K

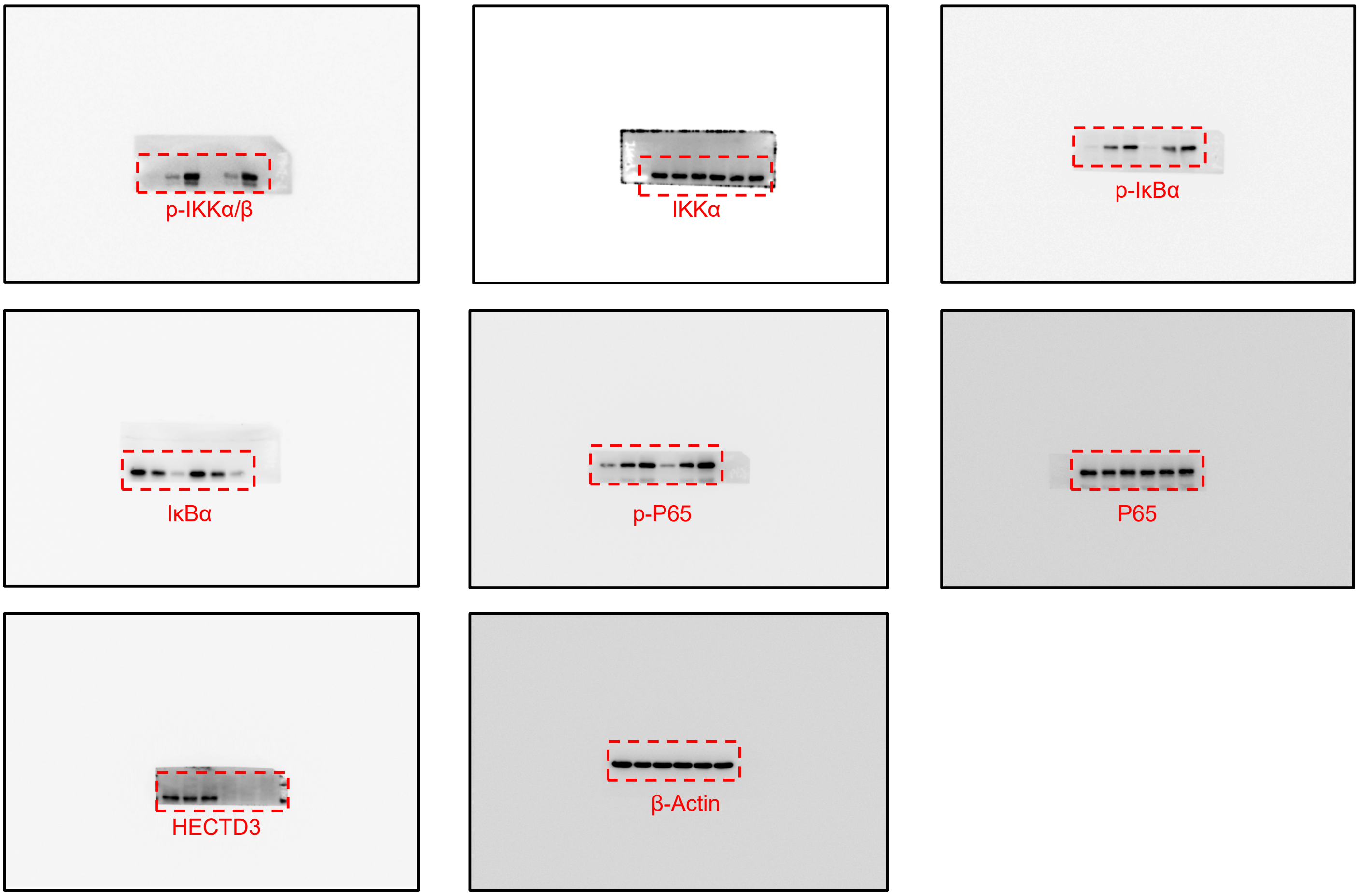
p-IKKα/β
IKKα
p-IκBα
IκBα
p-P65
P65
HECTD3
β-Actin

Fig.2E

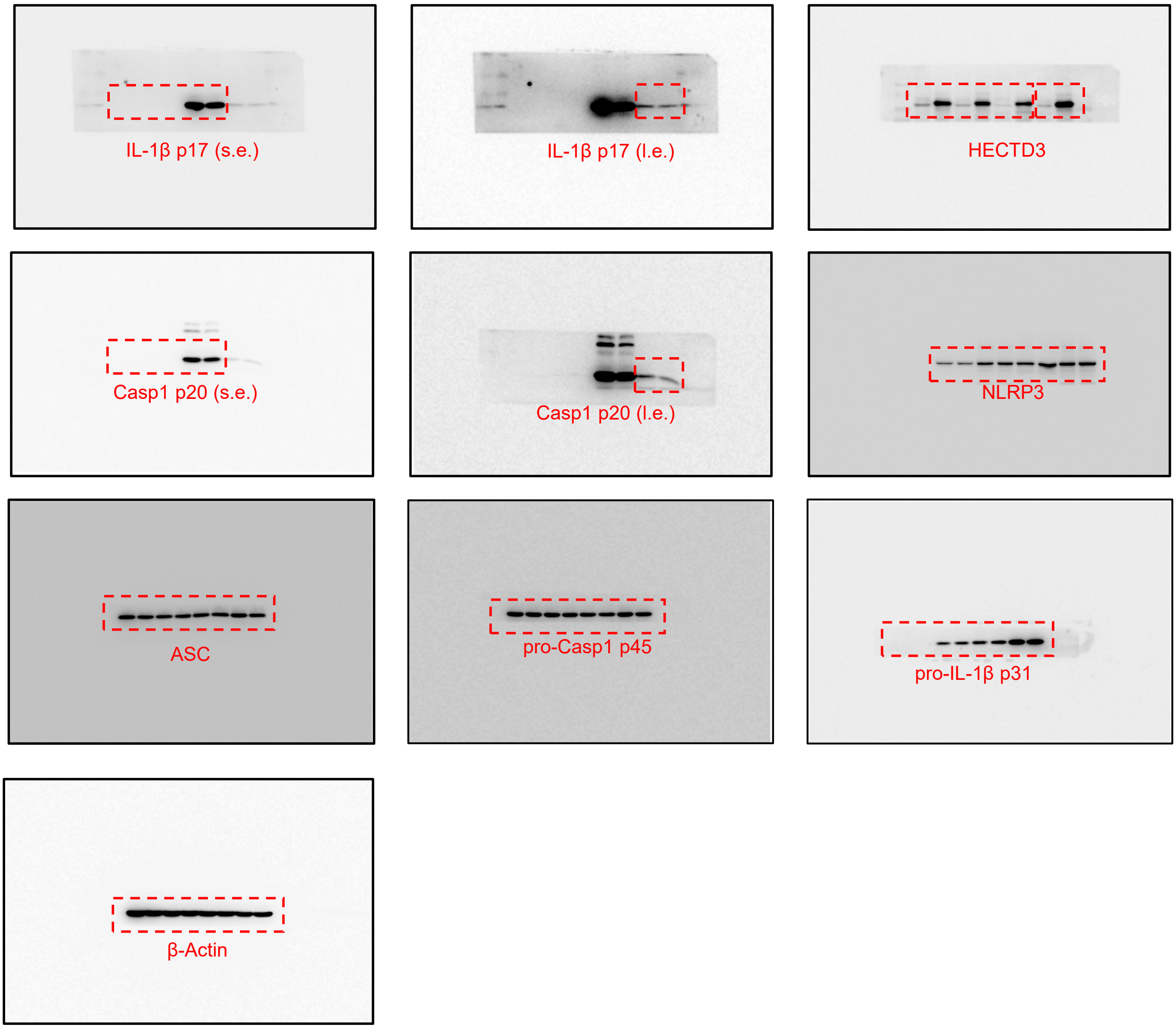
IL-1β p17 (s.e.)
IL-1β p17 (l.e.)
HECTD3
Casp1 p20 (s.e.)
Casp1 p20 (l.e.)
NLRP3
ASC
pro-Casp1 p45
pro-IL-1β p31
β-Actin

Fig.2H

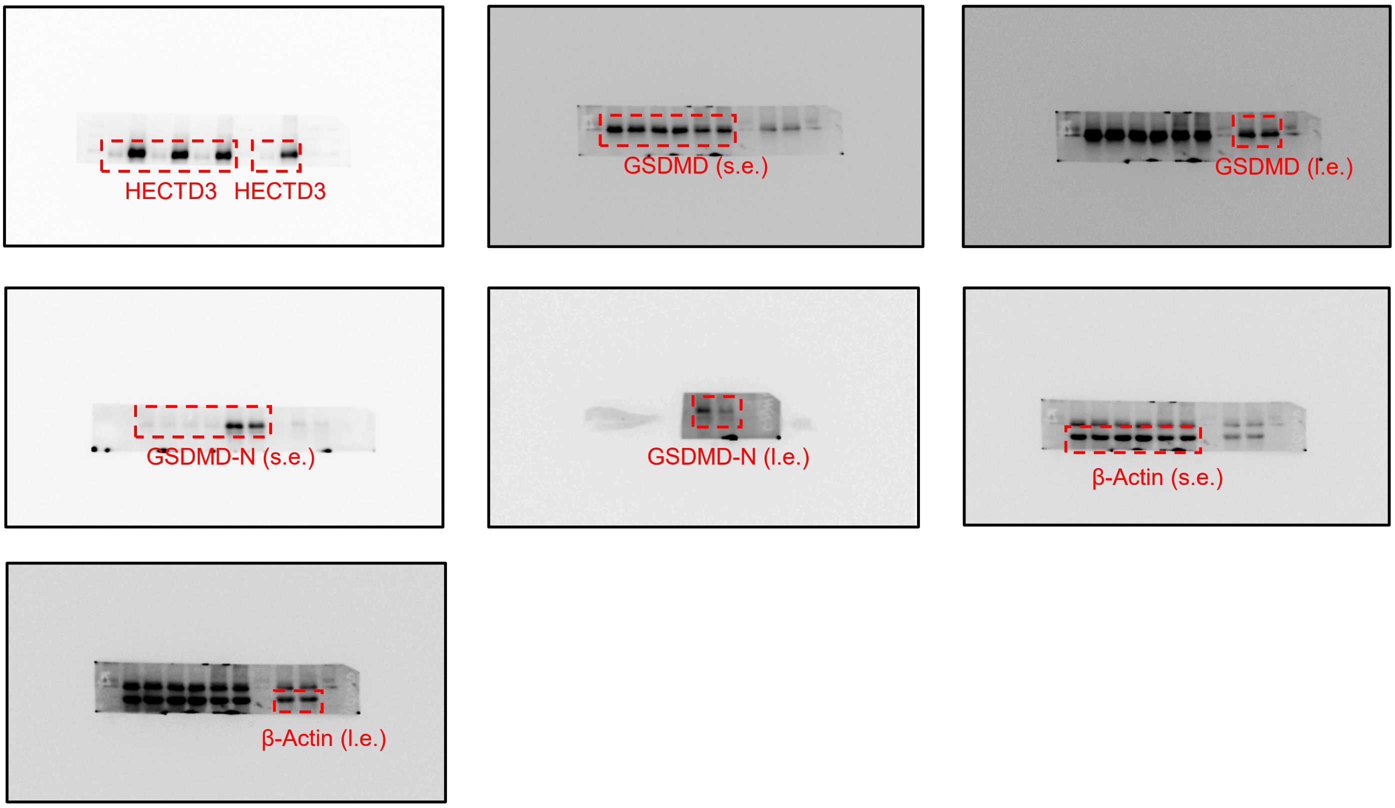
HECTD3
HECTD3
GSDMD (s.e.)
GSDMD (l.e.)
GSDMD-N (s.e.)
GSDMD-N (l.e.)
β-Actin (s.e.)
β-Actin (l.e.)

Fig.2I

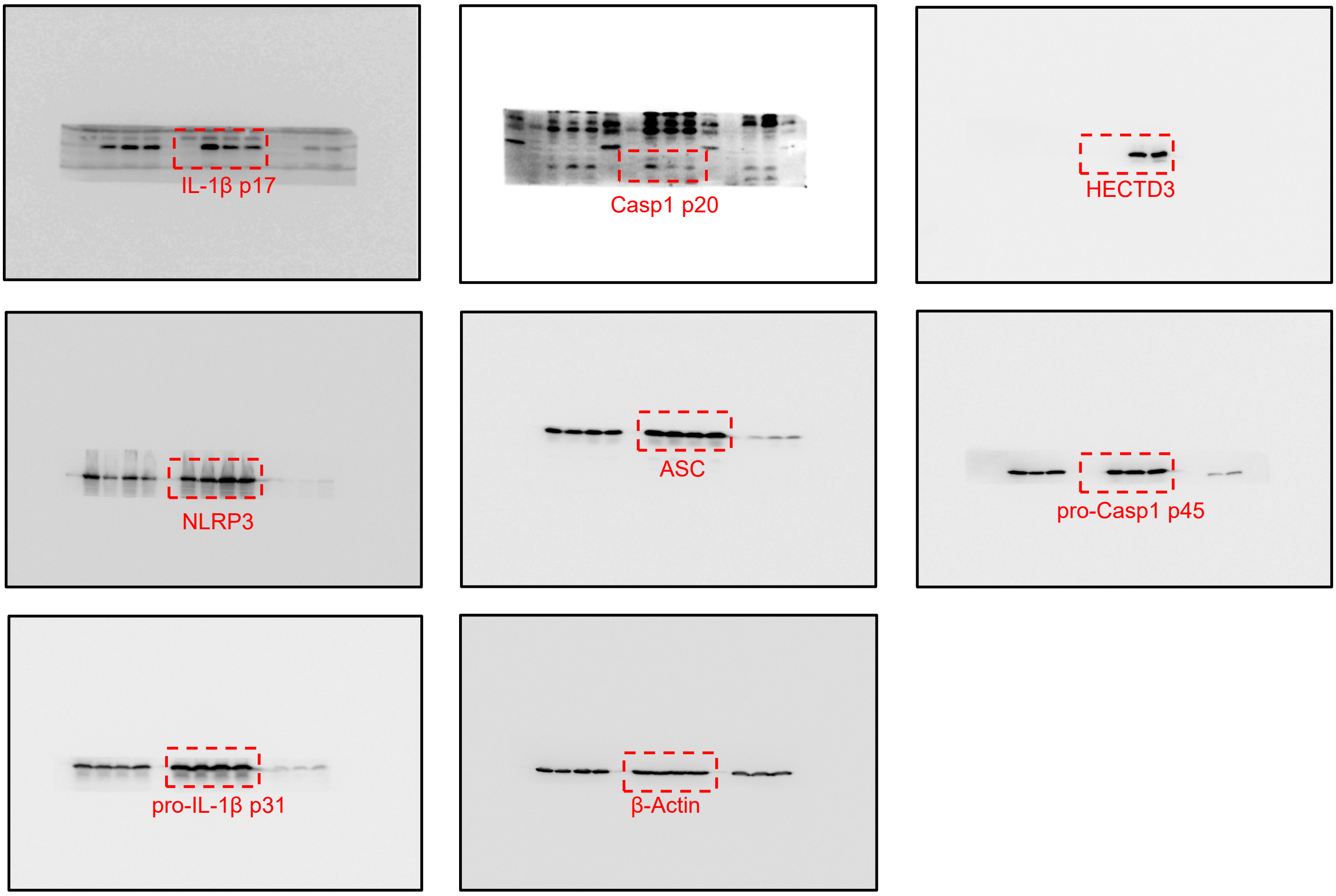
IL-1β p17
Casp1 p20
HECTD3
NLRP3
ASC
pro-Casp1 p45
pro-IL-1β p31
β-Actin

Fig.2K

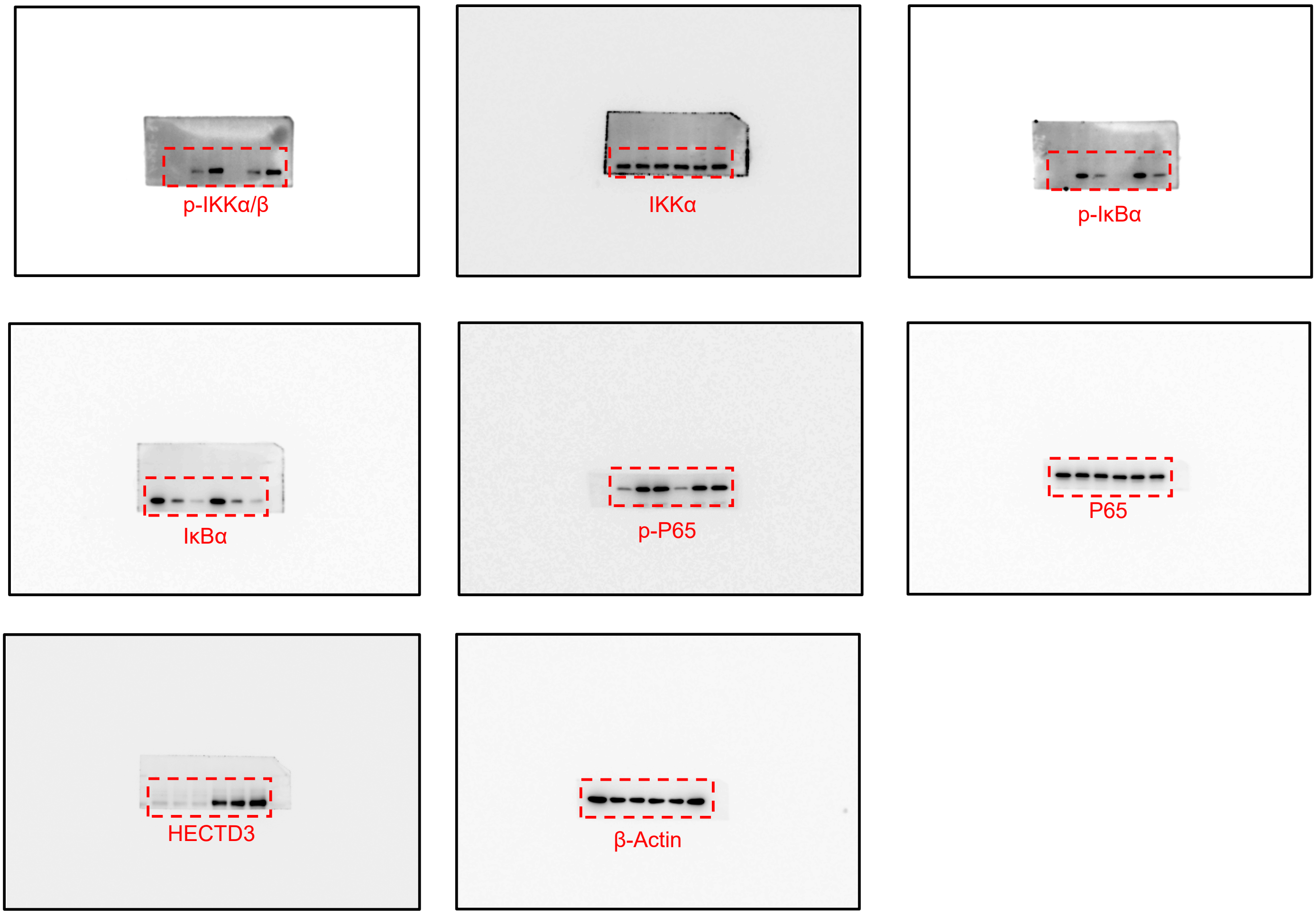
p-IKKα/β
IKKα
p-IκBα
IκBα
p-P65
P65
HECTD3
β-Actin

Fig.3B

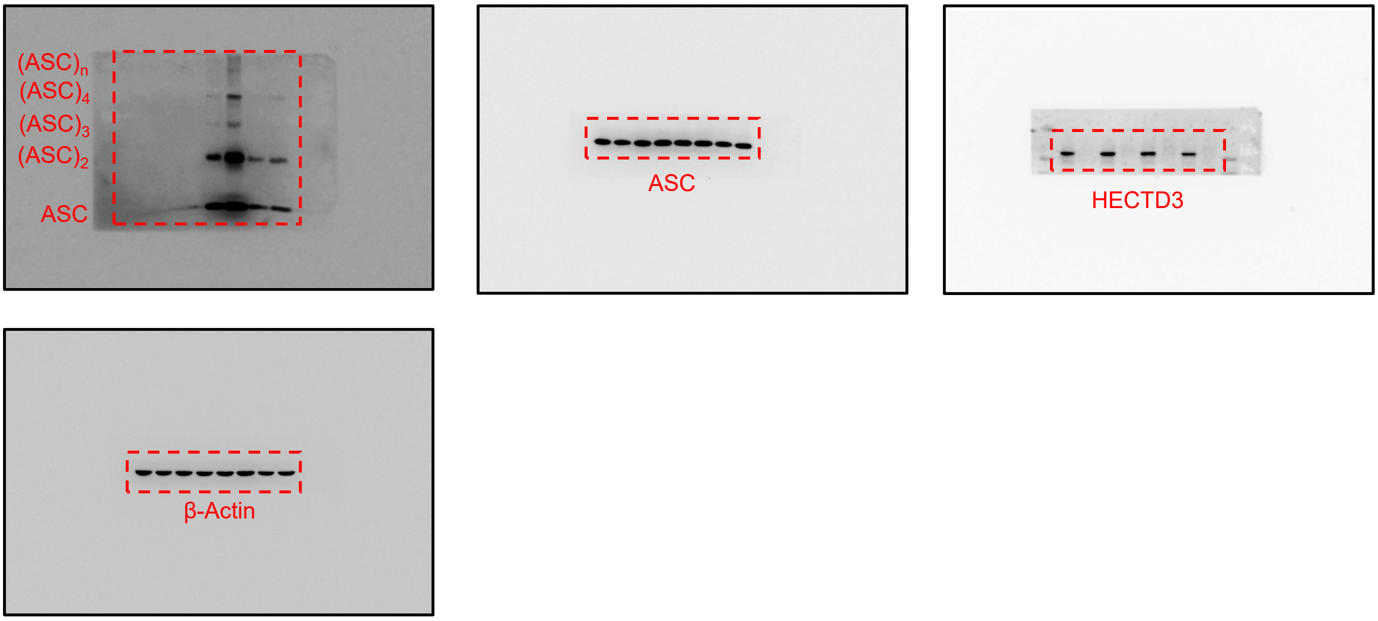

(ASC)n
(ASC)4
(ASC)3
(ASC)2
ASC
ASC
HECTD3
β-Actin

Fig.3C

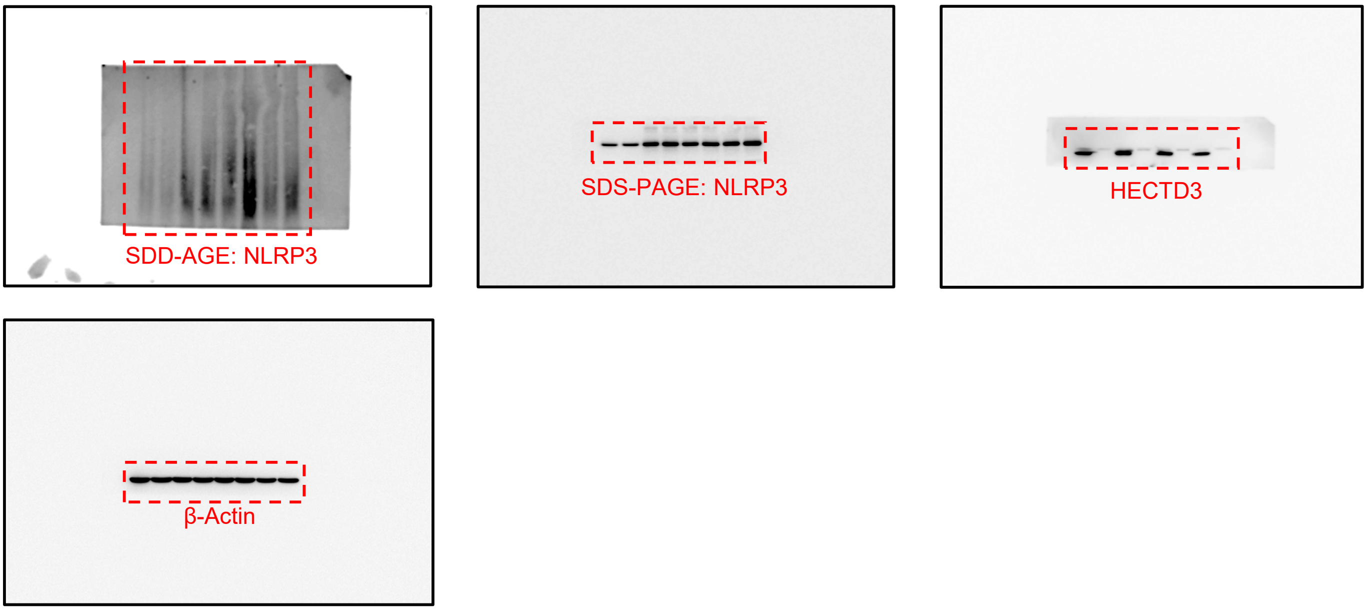

SDD-AGE: NLRP3
SDS-PAGE: NLRP3
HECTD3
β-Actin

Fig.3D

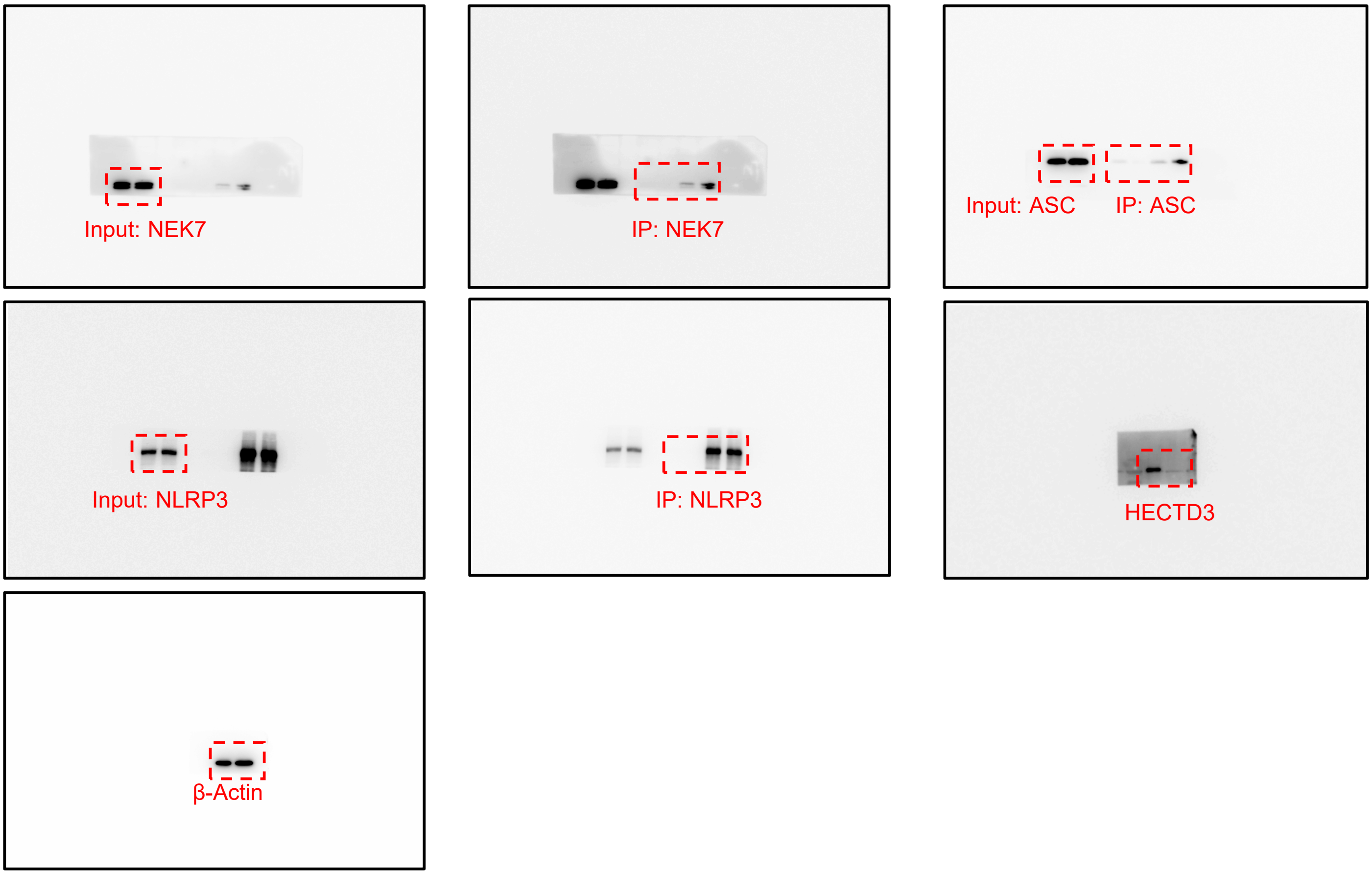
Input: NEK7
IP: NEK7
Input: ASC
IP: ASC
Input: NLRP3
IP: NLRP3
HECTD3
β-Actin

Fig.3E

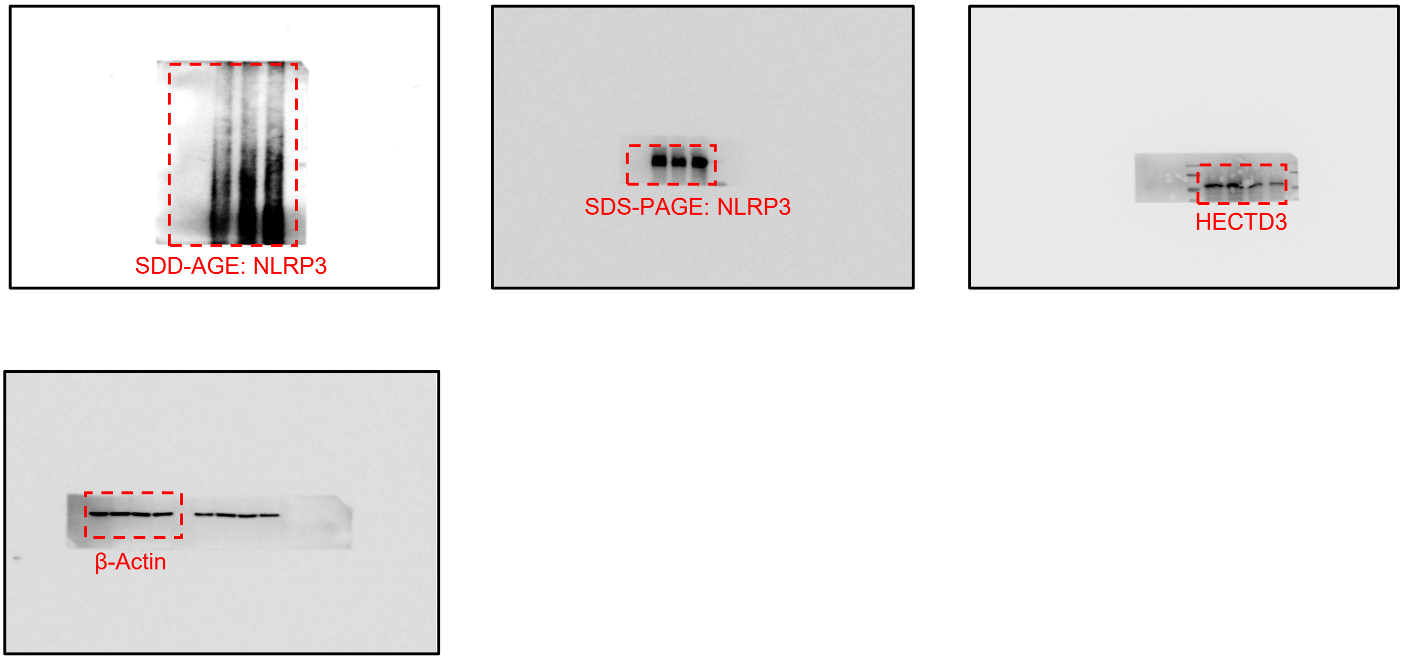
SDD-AGE: NLRP3
SDS-PAGE: NLRP3
HECTD3
β-Actin

Fig.3F

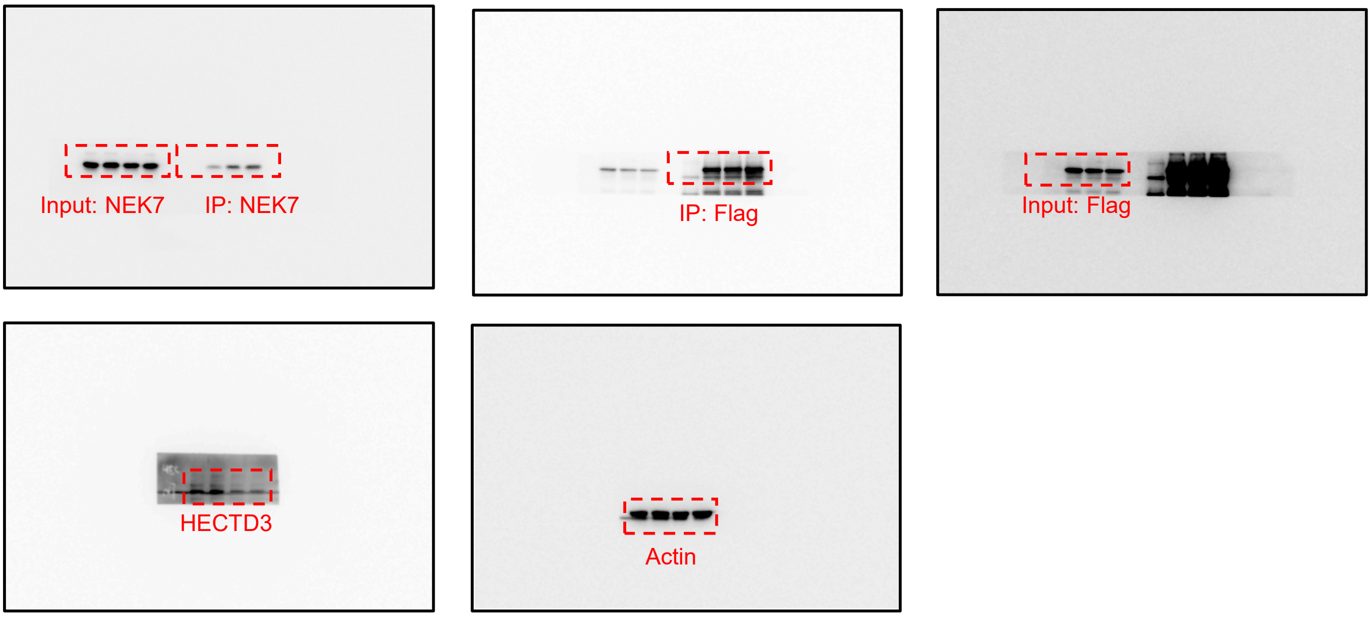
Input: NEK7
IP: NEK7
IP: Flag
Input: Flag
HECTD3
Actin

Fig.3G

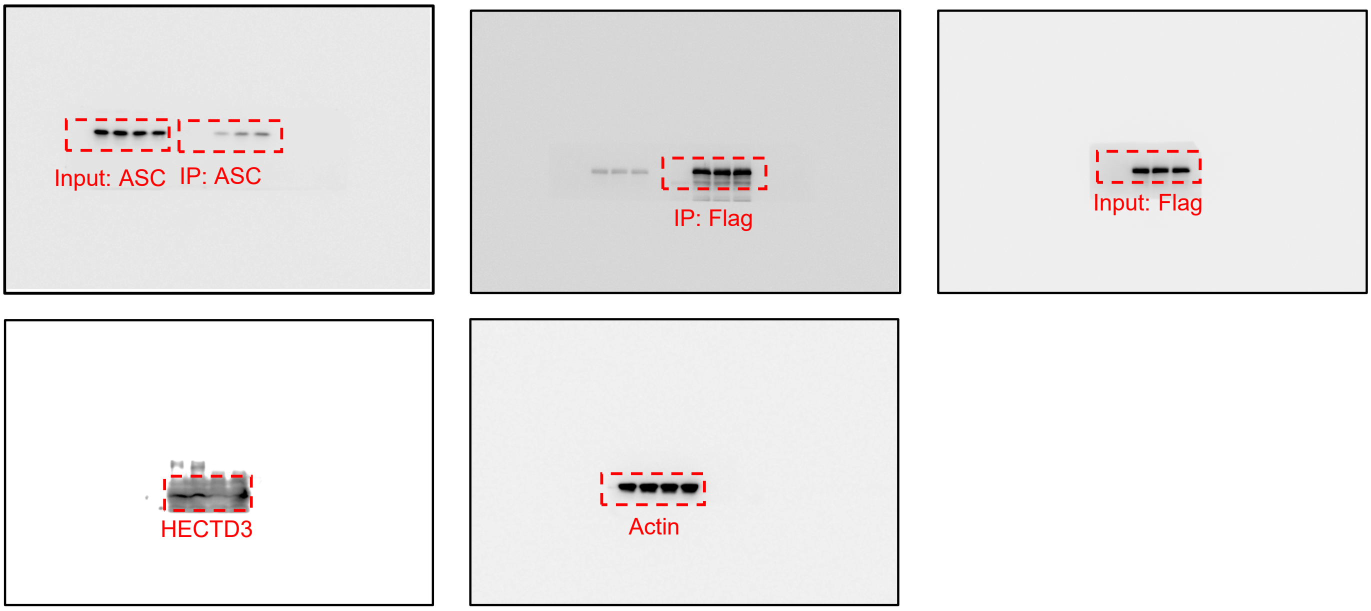
Input: ASC
IP: ASC
IP: Flag
Input: Flag
HECTD3
Actin

Fig.4B

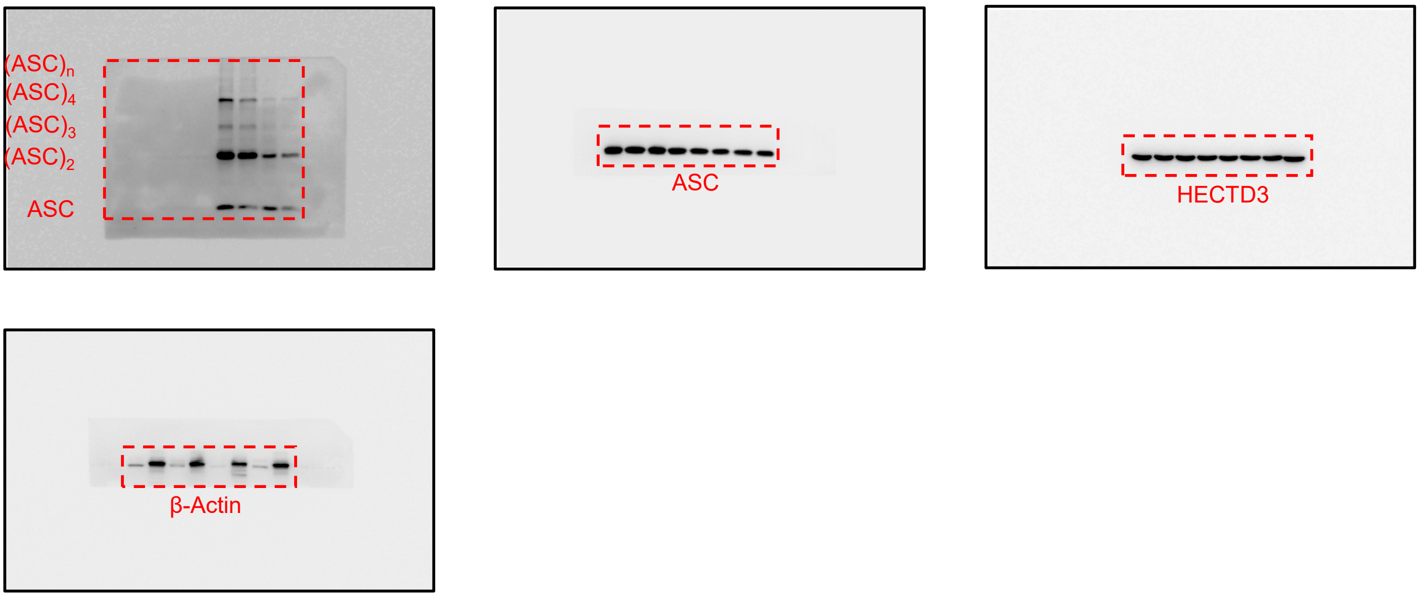
(ASC)$_n$
(ASC)$_4$
(ASC)$_3$
(ASC)$_2$
ASC
ASC
HECTD3
β-Actin

Fig.4C

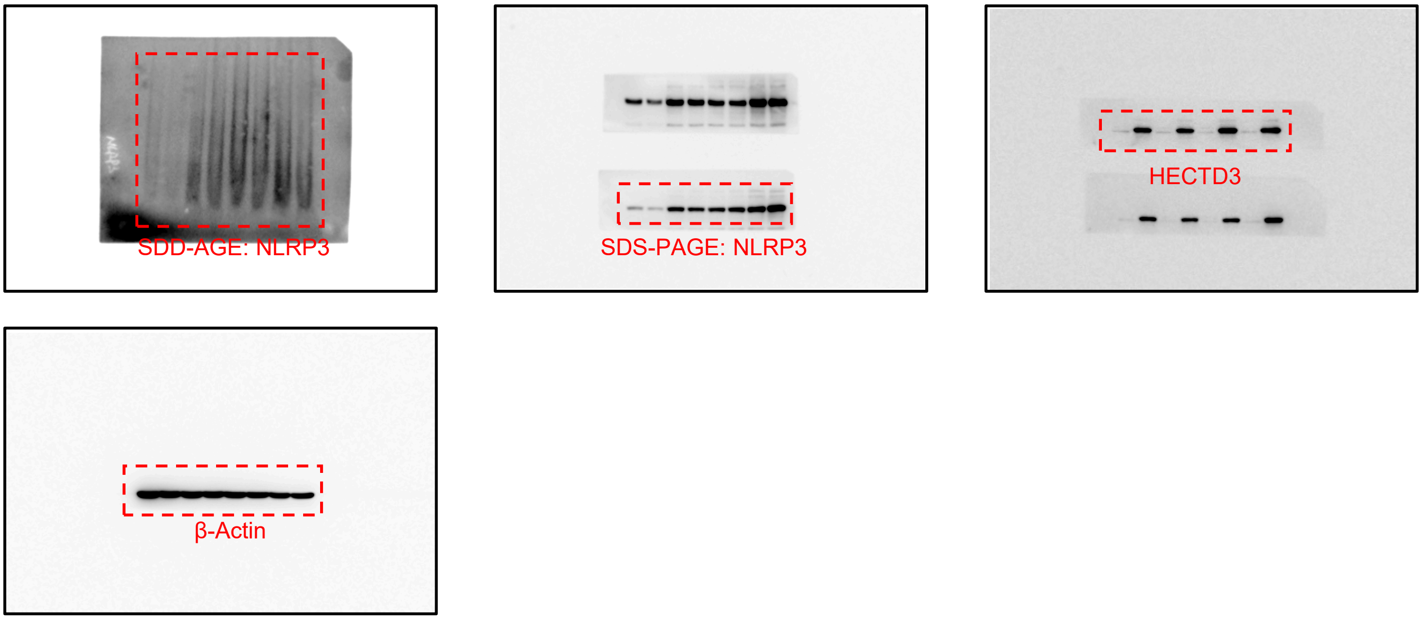
SDD-AGE: NLRP3
SDS-PAGE: NLRP3
HECTD3
β-Actin

Fig.4D

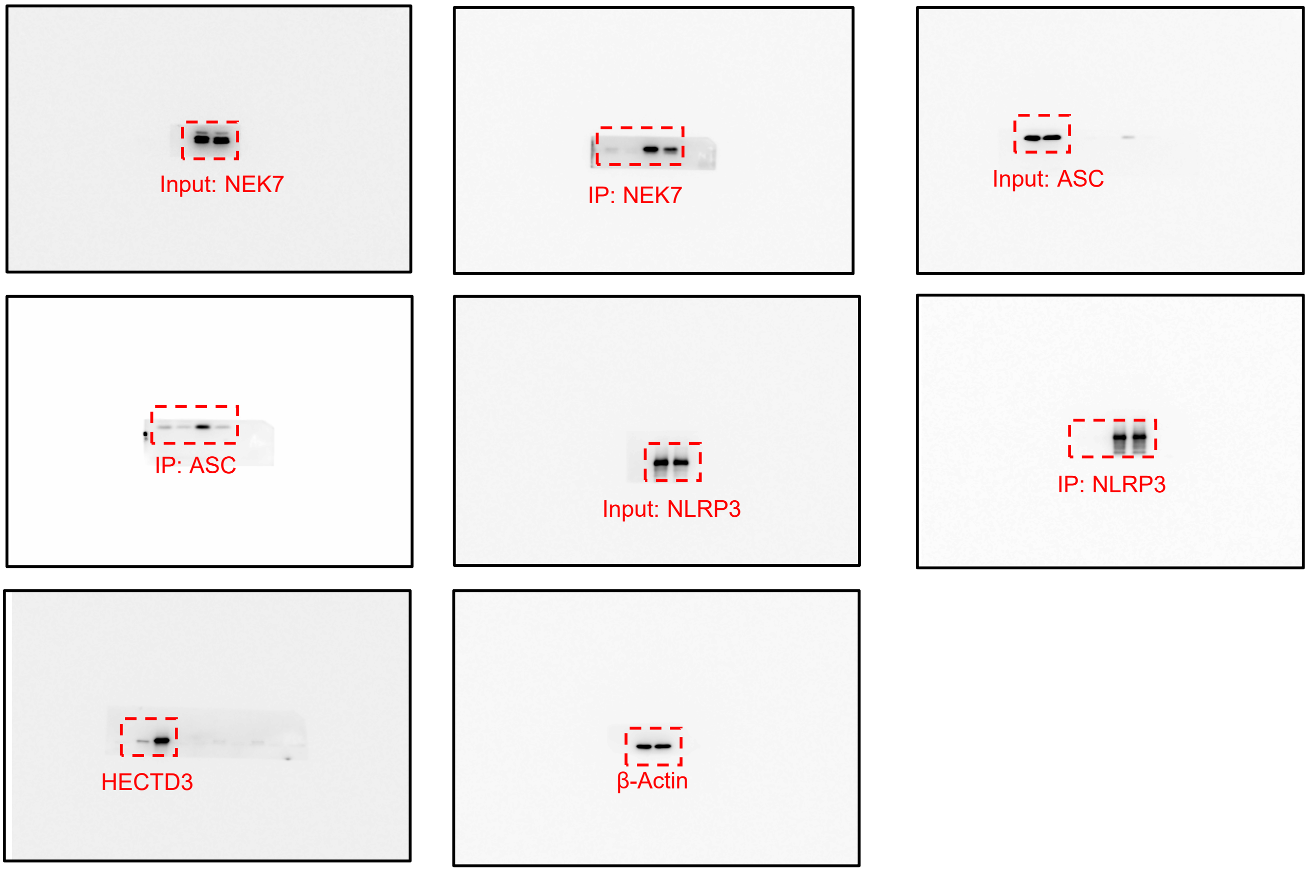
Input: NEK7
IP: NEK7
Input: ASC
IP: ASC
Input: NLRP3
IP: NLRP3
HECTD3
β-Actin

Fig.4E

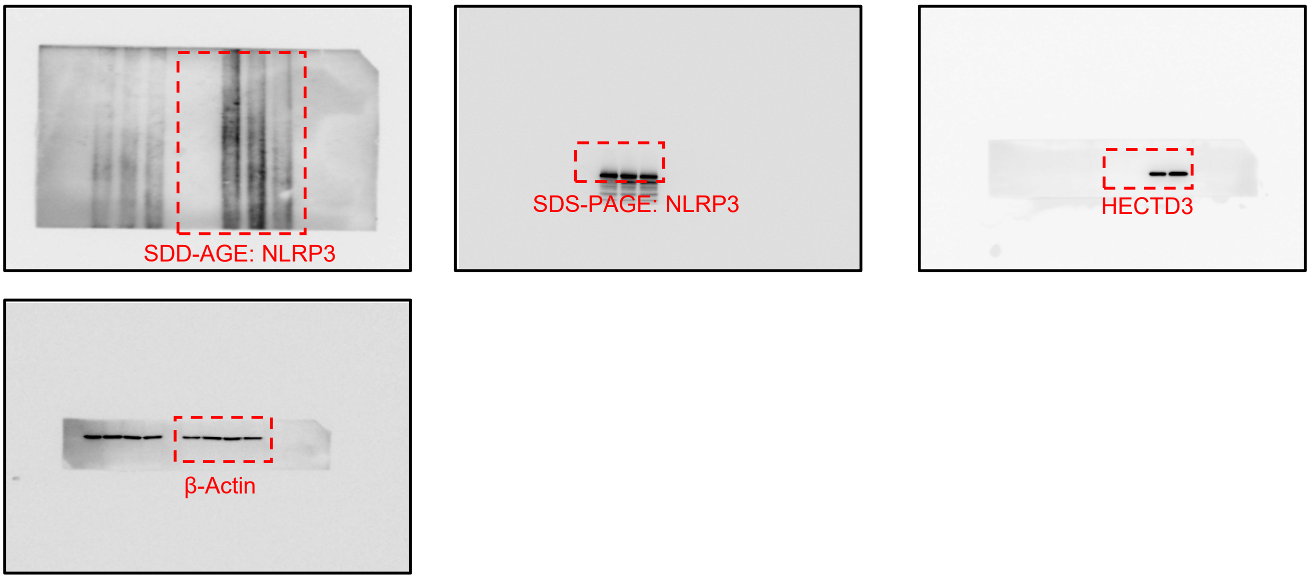
SDD-AGE: NLRP3
SDS-PAGE: NLRP3
HECTD3
β-Actin

Fig.4F

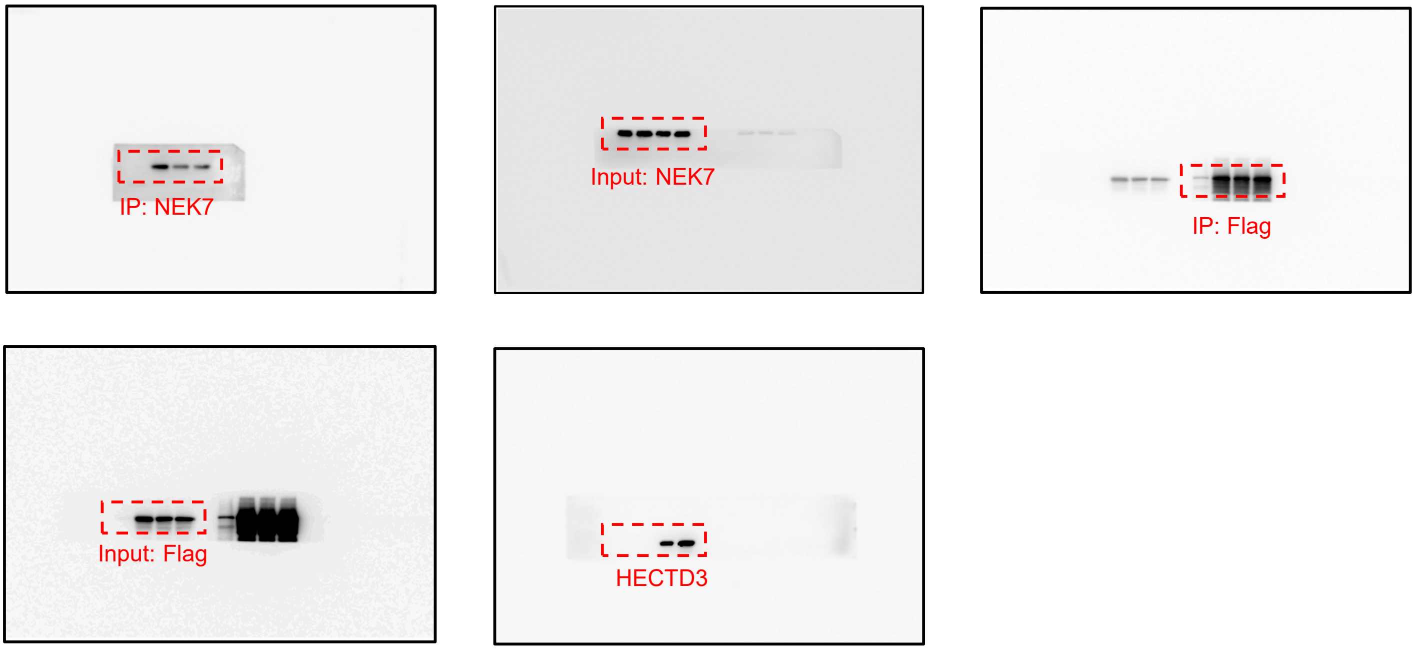
IP: NEK7
Input: NEK7
IP: Flag
Input: Flag
HECTD3

Fig.4G

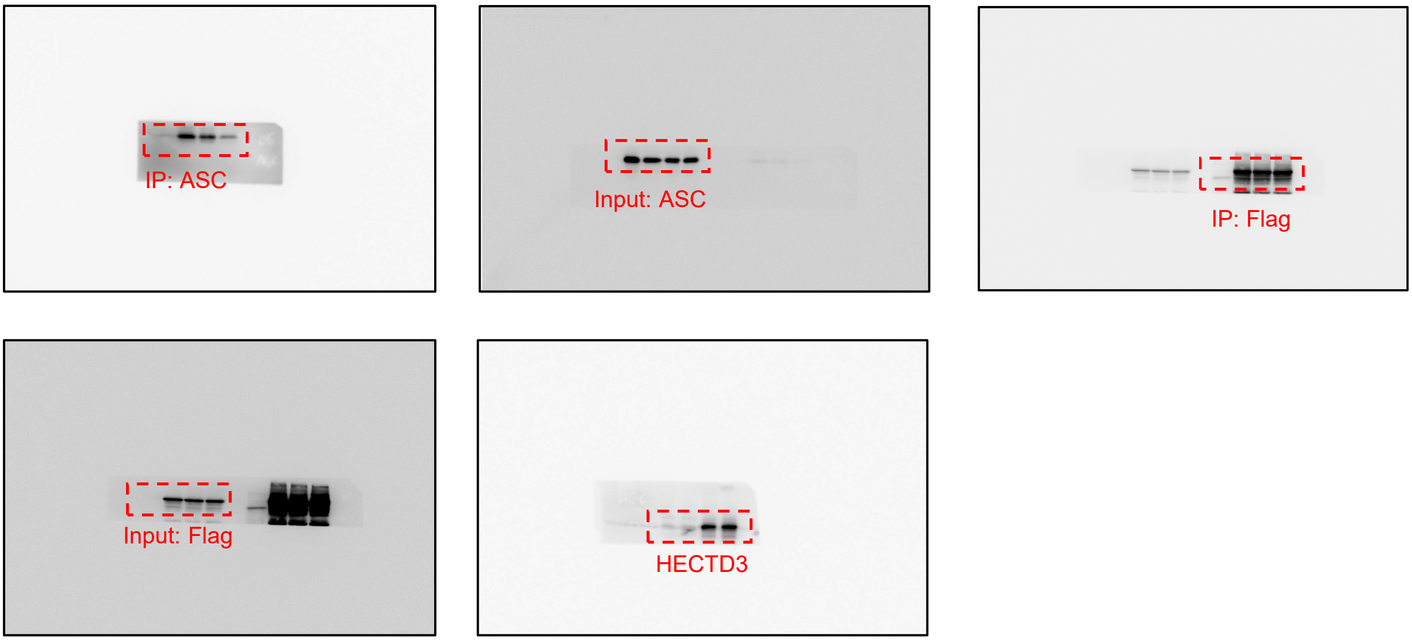
IP: ASC
Input: ASC
IP: Flag
Input: Flag
HECTD3

Fig.5A

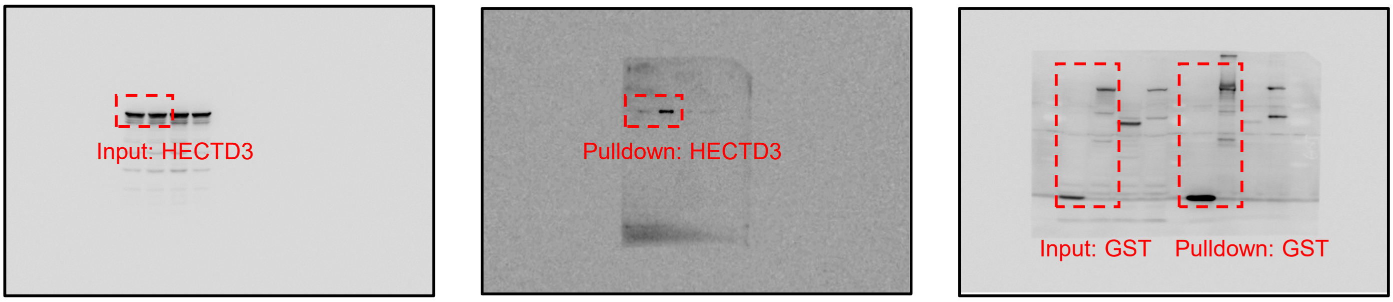

Fig.5B

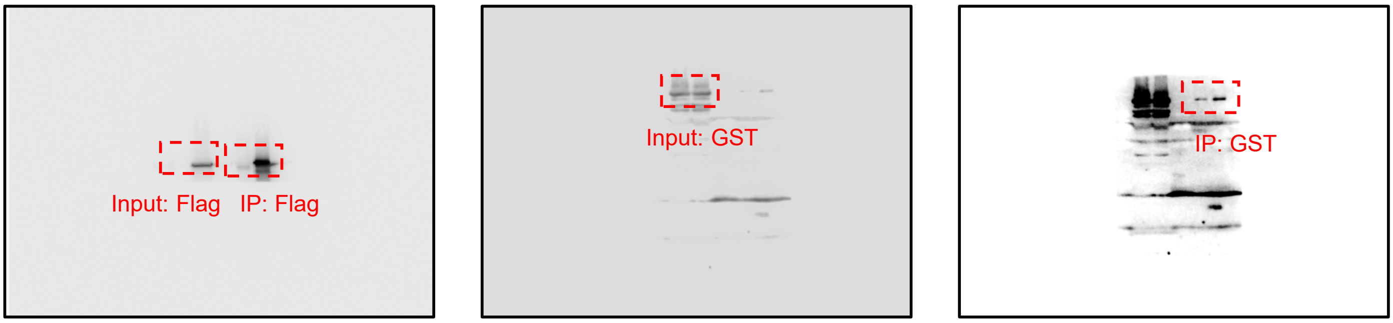

Fig.5C

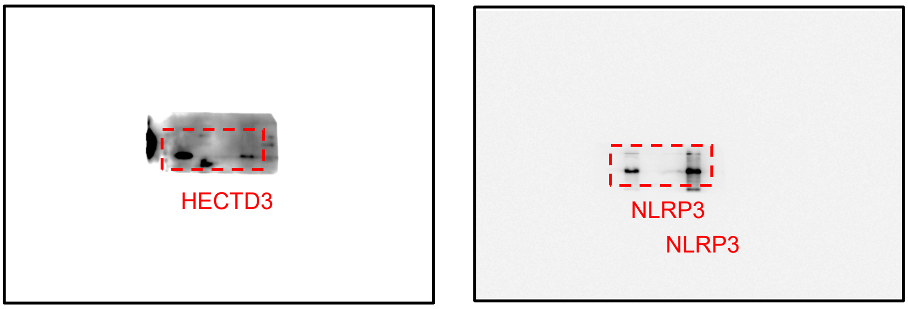

Fig.5G

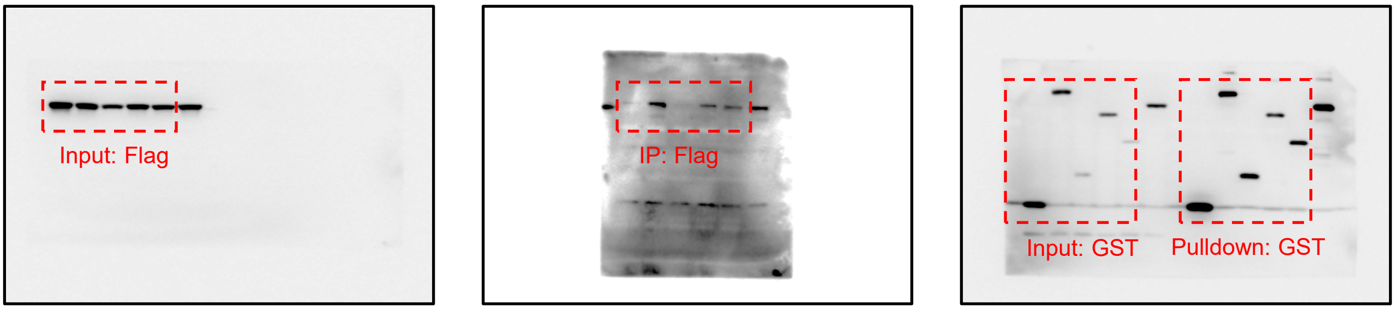

Fig.5H

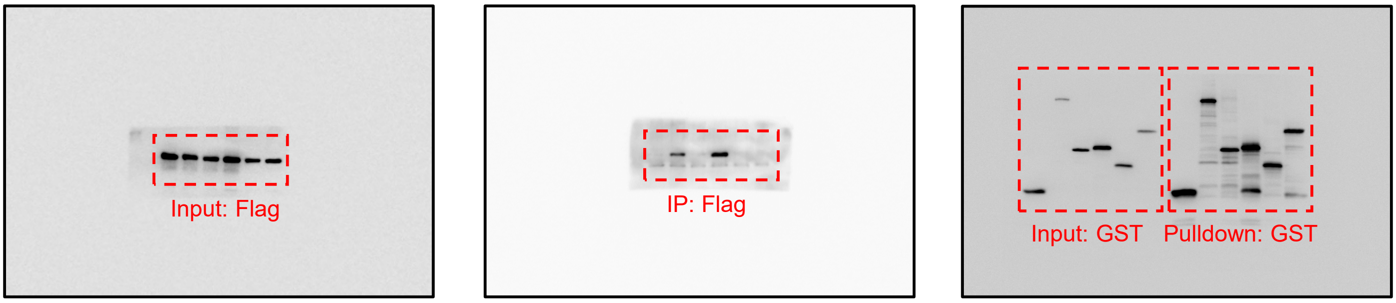

Fig.5I

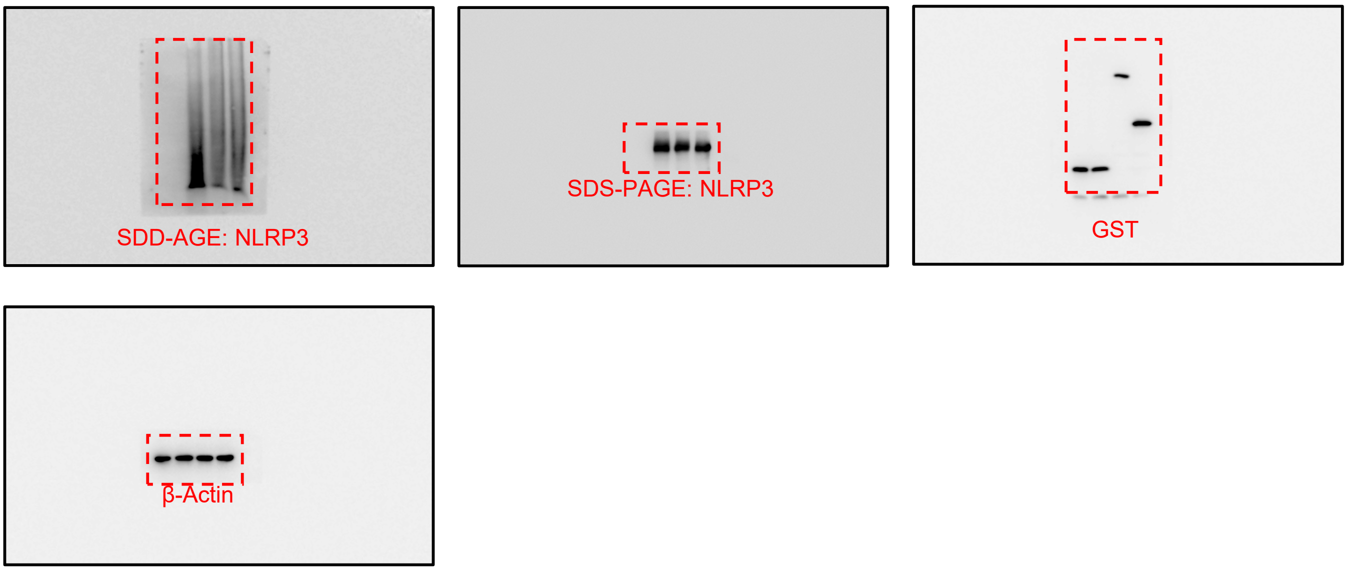
SDD-AGE: NLRP3
SDS-PAGE: NLRP3
GST
β-Actin

Fig.5J

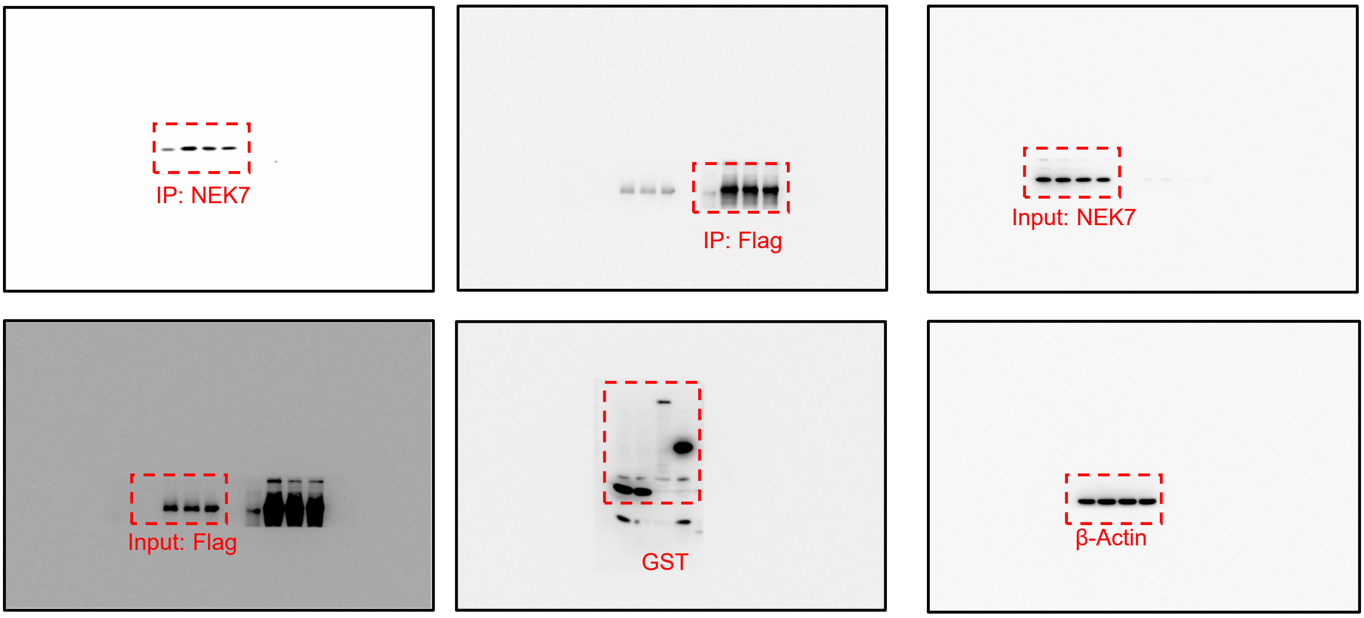
IP: NEK7
IP: Flag
Input: NEK7
Input: Flag
GST
β-Actin

Fig.5K

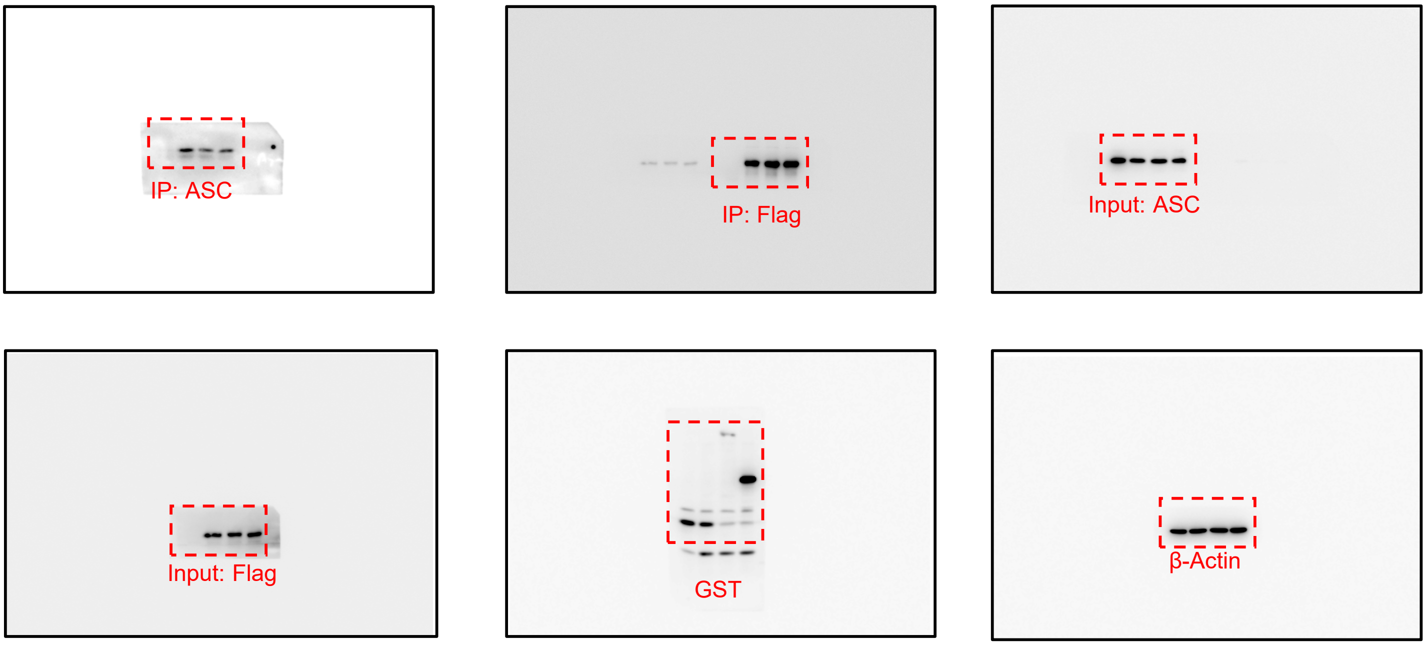
IP: ASC
IP: Flag
Input: ASC
Input: Flag
GST
β-Actin

Fig.5L

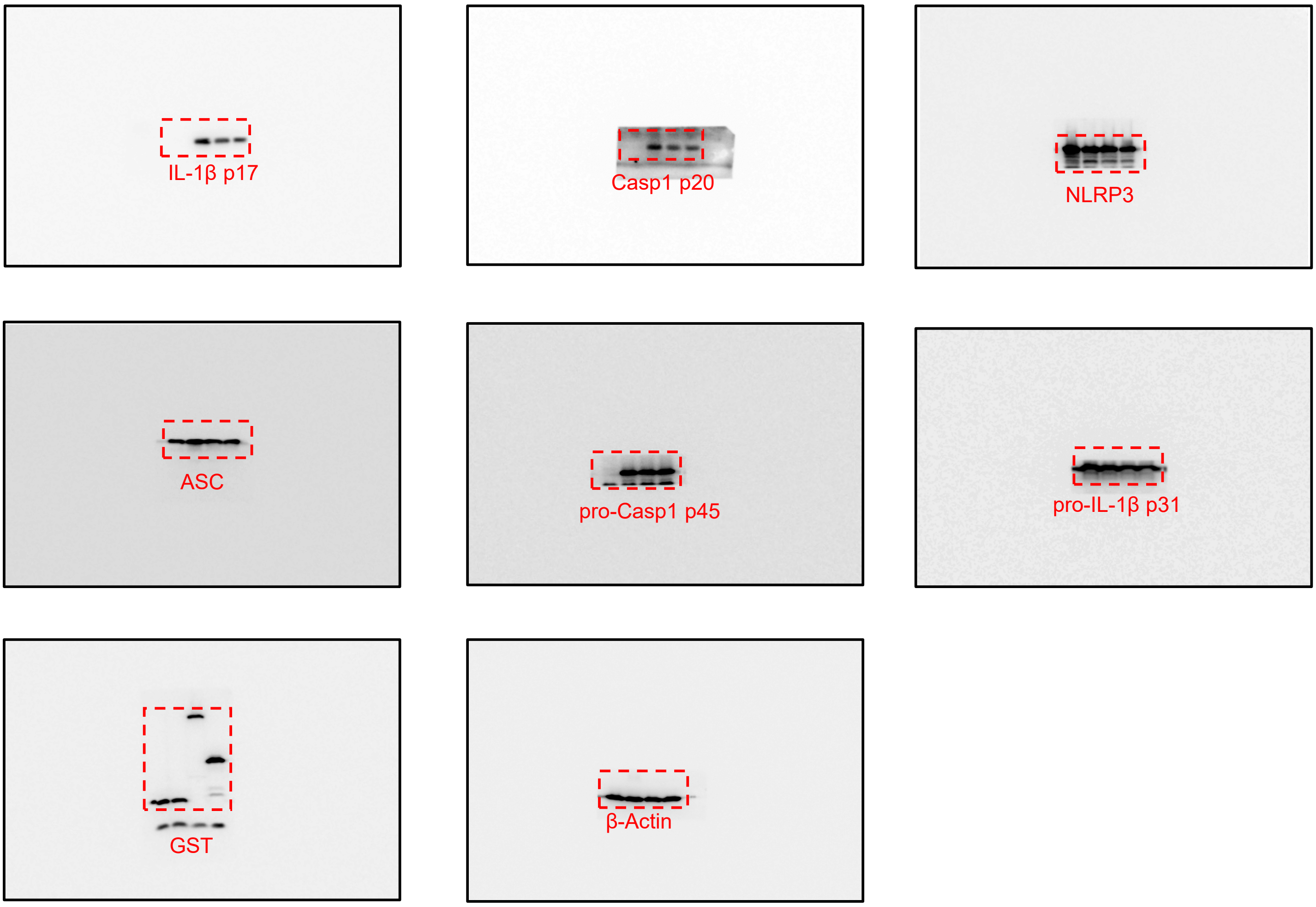
IL-1β p17
Casp1 p20
NLRP3
ASC
pro-Casp1 p45
pro-IL-1β p31
GST
β-Actin
